# Atomic structure and phason modes of the Sc–Zn icosahedral quasicrystal

**DOI:** 10.1107/S2052252516007041

**Published:** 2016-06-14

**Authors:** Tsunetomo Yamada, Hiroyuki Takakura, Holger Euchner, Cesar Pay Gómez, Alexei Bosak, Pierre Fertey, Marc de Boissieu

**Affiliations:** a Institute of Multidisciplinary Research for Advanced Materials (IMRAM), Tohoku University, Miyagi 980-8577, Japan; b Division of Applied Physics, Faculty of Engineering, Hokkaido University, Hokkaido 060-8628, Japan; c Institute of Materials Science and Technology, Vienna University of Technology, Vienna 1040, Austria; d Department of Chemistry, Ångström Laboratory, Uppsala University, Uppsala 751 21, Sweden; e ESRF – The European Synchrotron, Grenoble F-38043, France; f Synchrotron SOLEIL, Gif-sur-Yvette F-91192, France; g Université Grenoble Alpes, SIMAP, Grenoble F-38000, France; h CNRS, SIMAP, Grenoble F-38000, France

**Keywords:** quasicrystals, superspace crystallography, structure analysis, phasons, X-ray diffuse scattering

## Abstract

The detailed atomic structure of the binary icosahedral (i) ScZn_7.33_ quasicrystal has been investigated by means of high-resolution synchrotron single-crystal X-ray diffraction and absolute scale measurements of diffuse scattering. The average atomic structure has been solved using the measured Bragg intensity data based on a six-dimensional model that is isostructural to the i-YbCd_5.7_ one. The structure is described with a quasiperiodic packing of large Tsai-type rhombic triacontahedron clusters and double Friauf polyhedra (DFP), both resulting from a close-packing of a large (Sc) and a small (Zn) atom. The difference in chemical composition between i-ScZn_7.33_ and i-YbCd_5.7_ was found to lie in the icosahedron shell and the DFP where in i-ScZn_7.33_ chemical disorder occurs on the large atom sites, which induces a significant distortion to the structure units. The intensity in reciprocal space displays a substantial amount of diffuse scattering with anisotropic distribution, located around the strong Bragg peaks, that can be fully interpreted as resulting from phason fluctuations, with a ratio of the phason elastic constants *K*_2_/*K*_1_ = −0.53, *i.e.* close to a threefold instability limit. This induces a relatively large perpendicular (or phason) Debye–Waller factor, which explains the vanishing of ‘high-*Q*_perp_’ reflections.

## Abstract 2

The detailed atomic structure of the binary icosahedral ScZn_7.33_ quasicrystal has been investigated by means of high-resolution synchrotron single-crystal X-ray diffraction and absolute scale measurements of diffuse scattering.

## 1. Introduction   

Quasicrystals (QCs) exhibit a diffraction pattern with sharp Bragg reflections, as a signature of long-range order, yet with symmetries that are incompatible with three-dimensional translational symmetry (Shechtman *et al.*, 1984 ▸; Levine & Steinhardt, 1984 ▸). The structure of quasicrystals is best described in the framework of the superspace formalism, where the quasicrystal is embedded in a higher dimension (see Janot, 1992 ▸; Janssen *et al.*, 2007 ▸, for an introduction). In the case of icosahedral (i) QCs, the superspace periodic lattice is defined in six-dimensions (six-dimensional) and it is decomposed into two three-dimensional subspaces: the parallel (or physical or external) space, 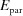, and the perpendicular (or complementary or internal) space, 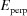. The quasiperiodic atomic arrangement in 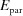 is obtained by an irrational cut through the six-dimensional periodic lattice decorated with three-dimensional objects, lying in 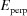, called occupation domains (ODs) or atomic surfaces.

The description of the atomic structure of the binary i-YbCd_5.7_ QC (Tsai *et al.*, 2000 ▸; Guo *et al.*, 2000 ▸) is now available in detail and with a level of accuracy close to what is achieved for periodic complex metallic alloys (Takakura *et al.*, 2007 ▸). This was made possible by the binary character of the sample and a subtle six-dimensional refinement procedure on large data sets obtained by X-ray synchrotron radiation. The knowledge of the detailed atomic structures of the 2/1 cubic approximants (cAPs) to the QCs, in addition to those of the common 1/1 cAPs, whose chemical compositions are close to i-YbCd_5.7_, also helped to build the complicated ODs for the six-dimensional structure model of i-YbCd_5.7_ (Tsai *et al.*, 2000 ▸; Gómez & Lidin, 2001 ▸; Takakura *et al.* (2001*a* ▸; Gómez & Lidin, 2003 ▸; Takakura *et al.*, 2007 ▸).

**Figure 1:**
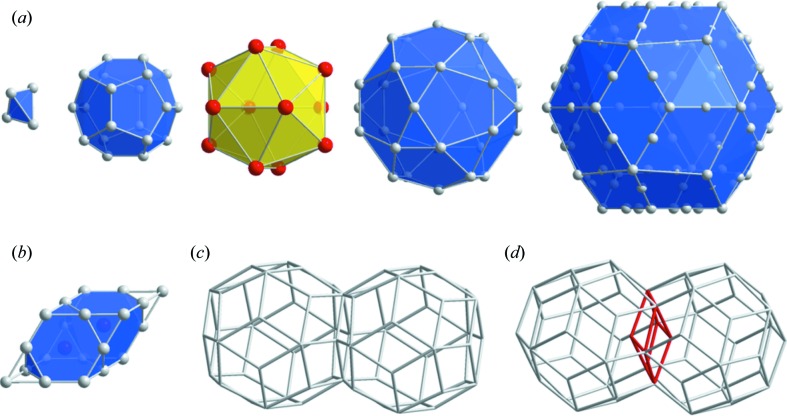
Building blocks for the i-YbCd_5.7_ QC model. (*a*) Successive shells of the Tsai-type rhombic triacontahedron (RTH) cluster. The radius of the RTH cluster is 7.8 Å along the twofold direction. (*b*) Double Friauf polyhedron (DFP) with edge length about 5.7 Å. Two different RTH cluster connections, (*c*) *b*-linkage, (*d*) *c*-linkage.

The i-YbCd_5.7_ structure is described as a close packing of large (Yb) and small (Cd) atoms, arranged in successive shells. Two basic clusters are necessary to describe the structure: a large Tsai-type rhombic triacontahedron (RTH) cluster and a double Friauf polyhedron (DFP). The large RTH cluster is built up from the following successive atomic shells: a central Cd_4_ tetrahedron, a Cd_20_ dodecahedron, a Yb_12_ icosahedron, a Cd_30_ icosidodecahedron and a decorated Cd_92_ RTH (see Fig. 1 ▸). All together the RTH cluster contains 158 atoms and has a radius of ∼ 7.8 Å along its twofold direction. The RTH clusters are located at so-called 12-fold vertices of a three-dimensional Ammann–Kramer–Neri (AKN) tiling proposed by Henley (1986 ▸) and connected along their twofold axis by sharing a face (*b*-linkage) and along their threefold axis where they overlap and define an oblate rhombohedron (*c*-linkage; see Figs. 1 ▸ *c* and *d*). The DFP can be viewed as a prolate rhombohedron, one of the two building units of the three-dimensional AKN tilling, having an edge length of 5.7 Å with Cd atoms at the vertices and edge center positions, and two Yb atoms along the longer body diagonal in the same way as in the YbCd_2_ Laves phase (Palenzona, 1971 ▸). The DFPs fill interstitial space within the RTH cluster network and have their longer body diagonal oriented parallel to a threefold axis.

The central tetrahedron in the RTH is believed to play a crucial role in the structural stability of this type of iQCs, as it relaxes locally a frustration arising from the icosahedral symmetry. Indeed, studies of ScZn_6_, isostructural to the YbCd_6_ 1/1 cAP to i-YbCd_5.7_, showed that the Zn_4_ tetrahedron reorients at room temperature (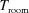) and its motion freezes along the [110] direction of the high-temperature phase below a critical temperature (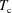) equal to ∼ 160 K (Ishimasa *et al.*, 2007 ▸; Yamada *et al.*, 2010 ▸, 2013 ▸; Euchner *et al.*, 2012 ▸). The low-temperature structural study has shown that the ordering of the Zn_4_ tetrahedron induces a large distortion to the successive shells, which can be understood by the close packing nature of the large (Sc) and small (Zn) atoms (Ishimasa *et al.*, 2007 ▸; Yamada *et al.*, 2013 ▸). In the 1/1 cAP, the Zn_4_ tetrahedron behaves as a single molecule and reorients constantly above 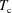. The same is true for the QCs, although no phase transition is observed, so that at 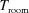 the tetrahedron reorients dynamically and the iQC structure displays an exceptional dynamical flexibility (Euchner *et al.*, 2012 ▸, 2013 ▸).

One of the interesting properties of QCs is related to phason modes, long wavelength fluctuations which exist in all aperiodic structures (Janssen *et al.*, 2007 ▸; de Boissieu *et al.*, 2007 ▸). It is related to the translational degree of freedom of the free energy expressed in 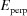 in the framework of hydrodynamic theory that leads to a long wavelength diffusive like excitation called a phason (Kalugin *et al.*, 1985 ▸; Bak, 1985*a* ▸,*b* ▸; Lubensky *et al.*, 1985 ▸). This fluctuation gives rise to phason diffuse scattering (PDS), located in the close vicinity of Bragg peaks and known to have a characteristic anisotropic shape (Jarić & Nelson, 1988 ▸; Widom, 1991 ▸; Ishii, 1992 ▸). So far, the PDS has been observed in all iQC phases for which measurements have been carried out (de Boissieu, 2012 ▸). However, its microscopic origin is still an open question.

One of the most challenging questions that remains unanswered is the mechanism that leads to the evolution of the long-range aperiodic order. Can a QC be grown with local rules? What are the respective contributions of the entropic and energetic terms? Are phason fluctuations and their associated entropy playing a crucial role in stabilizing the long-range aperiodic order? These are some of the questions, which are far from being understood, and for which experimental evidence is lacking.

Recently, a new iQC phase has been discovered in the Sc–Zn binary system and its chemical composition is given by ScZn_7.33_ (Canfield *et al.*, 2010 ▸). The atomic structure of this phase is of great interest because of the following reasons: (1) i-ScZn_7.33_ is one of the few binary iQCs, whose structure is supposed to be isostructural to those of i-YbCd_5.7_ and i-Ca_15_Cd_85_ (Tsai *et al.*, 2000 ▸; Guo *et al.*, 2000 ▸), however, its chemical composition significantly deviates from the latter ones (Canfield *et al.*, 2010 ▸). (2) It shows a large amount of X-ray diffuse scattering phenomena, elongated along directions parallel to the threefold axes, around strong Bragg peaks (Canfield *et al.*, 2010 ▸; Goldman *et al.*, 2011 ▸). (3) It is supposed to be the ‘parent’ phase of a number of iQC phases discovered in the ternary Sc–Zn–*M* systems (*M* = Mg, Mn, Fe, Co, Cu, Ag, Au, Pd, Pt) (Kaneko *et al.*, 2001 ▸; Kaneko & Ishimasa, 2002 ▸; Kashimoto *et al.*, 2003 ▸; Maezawa *et al.*, 2004 ▸; Lin & Corbett, 2004 ▸).

In this paper we present a detailed structural analysis of the binary i-ScZn_7.33_ QC. Whereas a structural analysis using Bragg reflections alone leads to a temporal and spatial average structure, the diffuse scattering yields information on the two-body correlation function. This is why we studied the i-ScZn_7.33_ phase using both Bragg diffraction and diffuse scattering measurements. The structural analysis using Bragg intensities yields a model with a significant amount of chemical disorder on the Sc sites. On the other hand, the large amount of observed diffuse scattering is fully accounted for by the PDS and a ratio of the phason elastic constants (*K*_2_/*K*_1_) close to a threefold instability limit. Combining those results we discuss the microscopic origins of the phason fluctuations and the mechanisms stabilizing the i-ScZn_7.33_ QC phase.

This paper is organized as follows. §2 gives the experimental details, and §3 the structural quality analysis by means of high-resolution X-ray scattering measurements. The results of the structure analysis of i-ScZn_7.33_ using synchrotron single-crystal X-ray diffraction data are presented in §4. The absolute scale measurements of the diffuse scattering and its interpretation in terms of the PDS are presented in §5. The results are compared with other iQCs, in particular to i-Sc–Zn–Mg. The last section is devoted to discussion and summary.

## 2. Experimental details   

### 2.1. Sample preparation   

**Figure 2:**
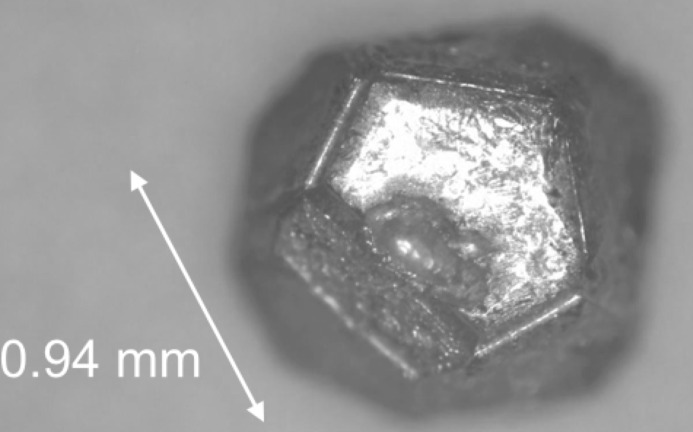
Optical microscope image of a single grained crystal of i-ScZn_7.33_.

Single grained samples of i-ScZn_7.33_ were obtained by a self-flux technique following the work by Canfield *et al.* (2010 ▸). The high purity elements of scandium (99.9 wt.%, Rare Metallic Co., Ltd) and zinc (99.9999 wt.%, Kojundo Chemical Laboratory Co., Ltd) with nominal composition Sc_4_Zn_96_ were placed in an alumina crucible sealed inside a silica tube under argon atmosphere. The elements were melted at 1093 K for 3 h and cooled to 893 K in 3 h and then slowly cooled at a rate of −1 K h^−1^ to 753 K. Finally, at this temperature the crystal grains were separated from the remaining zinc melt using a centrifuge technique. The morphology of the obtained single crystals is a pentagonal dodecahedron as shown in Fig. 2 ▸. Then the crystal grains were sealed inside a stainless steel tube under argon atmosphere and annealed at 473 K for 3 weeks, to remove quenched-in structural defects, such as point and topological defects.

### 2.2. Single-crystal X-ray diffraction experiments   

In order to determine the atomic structure of i-ScZn_7.33_, a single-crystal X-ray diffraction intensity measurement was performed on the CRISTAL beamline at the synchrotron SOLEIL. The incoming X-ray energy was selected by a double Si(111) monochromator and set to 25.55 keV. Two mirrors located after the monochromator focus the X-ray beam with a size equal to 0.12 × 0.45 mm. The single grained crystals of the annealed i-ScZn_7.33_ sample were crushed and a ∼60 µm piece of the single crystal was mounted on a cactus needle. A systematic data collection was carried out at 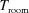 on a Kappa-type four-circle diffractometer equipped with an Atlas CCD detector (Rigaku Oxford Diffraction). A distance of 80 mm between the sample and the detector was set, and a φ rotation of 360°, with an oscillation width equal to 1.0°, was conducted. In order to collect intensity data with a high dynamic range, that is both strong and weak reflections, three successive measurements with attenuation factors equal to 1, 50 and 1000 were performed. The last one could prevent saturation of the CCD detector for all strong reflections. The data sets were processed using the software package *CrysAlisPro* (Agilent Technologies). The details of intensity data collection are given in the supporting information.

A high-resolution single-crystal X-ray diffraction experiment was performed on the French CRG D2AM beamline located on a bending magnet at the European Synchrotron Radiation Facility (ESRF). The incoming X-ray energy was selected by a double Si(111) monochromator located in between two mirrors, which suppress higher harmonics and focus the beam vertically. The incident energy was set to 9.3 keV, just below the Zn K-edge, to avoid Zn fluorescence. Two single-crystal samples, as-cast crystal and annealed crystal, both having polished faces normal to a fivefold axis with an approximate surface size of 1 × 1 mm^2^ were used. Each sample was mounted on a goniometer head and placed inside a beryllium chamber under vacuum to suppress air scattering. All measurements were performed at 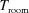 in reflection geometry on a seven-circle diffractometer equipped with a scintillation point detector. The distance from the sample to the detector was equal to 0.6028 m. This experimental configuration enabled a high dynamic range measurement, covering almost nine orders of magnitude. Systematic *Q*-scans were carried out along several high symmetry axes of icosahedral symmetry. The diffuse scattering intensity was put on an absolute scale, using the following standard normalization
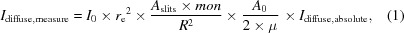
where 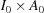 is the number of photons per monitor, μ is the linear absorption coefficient, 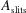 is the receiving slit area, *R* is the distance between sample and detector, 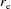 is the classical electron radius and *mon* is the monitored intensity of the incoming beam (Warren, 1969 ▸; Létoublon *et al.*, 2001*a* ▸; Francoual, 2006 ▸). The absolute scale measurement is of crucial importance if any comparison with other samples is to be made. Indeed, unlike integrated intensity, the diffuse scattering intensity is, for instance, strongly dependent on the receiving slit distance and apertures. All absolute scale data are presented hereafter in electron units per cubic ångström (e.u. Å^−3^) and can be converted into electron units per atom using the atomic density equal to 0.064 atom Å^−3^. In addition, supplemental X-ray diffraction data for the large reciprocal intensity maps were measured on the beamline ID23-1 at ESRF using a Pilatus6M detector (DECTRIS Ltd). The incident energy was set to 16 keV.

## 3. Structural quality: phason strain   

Its mosaic spread on one hand, and its correlation length measured by longitudinal scans on the other hand usually give an evaluation standard of the structural quality of a single crystal. Whereas the former can be related to the mosaic block distribution in the crystal, the latter is related to the defects distribution such as dislocations, leading to a Bragg peak broadening. For QCs the same is of course true, since QCs belong to a special class of crystals, but additional defects known as phason strain or phason strain distribution have to be taken into account when analysing the Bragg peak broadening (Lubensky *et al.*, 1986 ▸; Horn *et al.*, 1986 ▸; Socolar & Wright, 1987 ▸; Lubensky, 1988 ▸). In that case, the Bragg peak width (or shift) correlates with the perpendicular space component of the six-dimensional scattering vector, *Q* 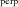 = 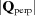 (see supporting information and references therein for a detailed explanation).

In order to evaluate the crystal quality of both as-cast and annealed i-ScZn_7.33_ samples, positions and profiles of Bragg peaks have been investigated using a high-resolution setup on the D2AM beamline at the ESRF. The sample mosaic spread was found to be 0.05°, which was about five times larger than the beam divergence. For the transverse scan we used another high-resolution setup, taking into account the sample mosaic, which gave a resolution expressed by its full width at half maximum (FWHM) equal to 2 × 10^−3^ Å^−1^ (a definition for the momentum transfer is 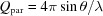, where θ and λ are the scattering angle and X-ray wavelength, respectively, and are employed throughout this paper) for Bragg peaks having a *Q* 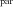 of the order 3 Å^−1^. For longitudinal scans, we reduced the vertical receiving slits, which were located at 600 mm, from 1 mm to 0.15 mm, leading to a resolution equal to 1 × 10^−3^ Å^−1^, corresponding to a correlation length equal to 6000 Å.

Hereafter, the length scales in reciprocal space are given in reciprocal lattice units (r.l.u.), using the indexing scheme proposed by Cahn *et al.* (1986 ▸). From the single-crystal diffraction data, the space group was determined to be 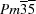 with an icosahedral lattice constant (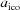) equal to 5.021 (3) Å. The conversion factor from r.l.u. to Å^−1^ is given by 2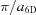 = 0.8848 (5) Å^−1^, where 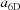 is the six-dimensional lattice constant equal to 7.101 (4) Å. Note that this definition is in particular important for the perpendicular space units since an arbitrary scale can be introduced. In the present indexing scheme the lattice constants in both 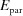 and 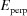 are set to be the same.

**Figure 3:**
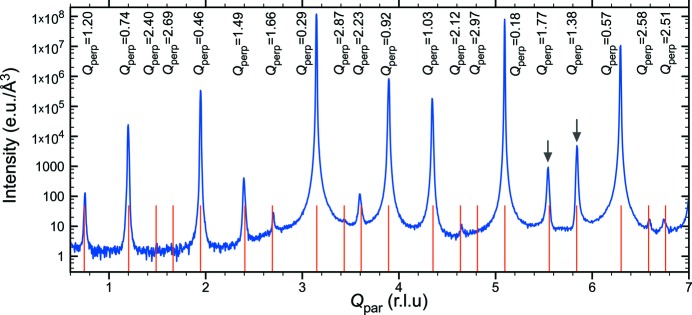
θ–2θ (longitudinal) scan measured along the twofold axis of the annealed i-ScZn_7.33_ sample. Vertical lines are the predicted positions of the Bragg reflections under icosahedral symmetry. The corresponding values of 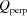 in r.l.u. are indicated. Two peaks indicated by arrows exhibit clear deviation from their ideal positions.

Fig. 3 ▸ shows a θ–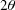 (longitudinal) scan of Bragg reflections on a twofold axis for the annealed i-ScZn_7.33_ sample. From the figure, we can see small but significant deviations of the Bragg peak positions from their ideal ones. Since the observed peak shifts are both positive and negative, systematic experimental errors such as displacement of the sample from the diffractometer axis can be ruled out. On the other hand, when plotted as a function of *Q* 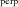 the deviation Δ*Q* 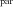 displays a linear dependence, which can be explained by the presence of a linear phason strain in the samples. In that case the shift Δ*Q* 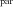 is obtained as the product between the phason strain matrix *M* and *Q* 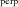. Because we used a projection of *Q* 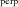 on the given symmetry axis, the peak shifts can be both positive and negative. Similar peak shifts of the Bragg peak were also observed for the as-cast sample.

The ‘magnitude’ of the phason strain can be evaluated based on the linear dependence of the peak shift as a function of *Q* 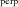 from which the slope α is extracted. Along the three- and fivefold axes the values of α are similar in both the as-cast and annealed samples and are equal to −7.8 × 10^−3^ and −3.6 × 10^−3^, respectively. Along the twofold direction the value of α in the as-cast sample is about four times larger than that of the annealed sample for which α equals 3.8 × 10^−3^. This indicates that the annealing did reduce the linear phason strain significantly, but could not suppress it.

The data we had at hand were not sufficiently accurate to determine the full phason strain matrix. However, since the largest phason strain amplitude occurs along the twofold axis, it can be postulated that the phason strain might be related to the symmetry breaking from icosahedral to 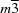 or lower. When the golden mean (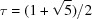) in the phason strain matrix is changed to a rational number, a periodic ‘rational’ AP is obtained (Elser & Henley, 1985 ▸; Ishii, 1989 ▸; Janssen, 1991 ▸; Gratias *et al.*, 1995 ▸). In that case, as a matter of comparison the α value for a 1/1 and a 2/1 cAP would be equal to −0.23 and 0.09, respectively.

It is also interesting to compare the results with those observed in the i-*R*–Zn–Mg (*R* = Y, Tb, Ho) phases for which a detailed study has been carried out (Létoublon *et al.*, 2000 ▸). Some samples did not show any Bragg peak shift, whereas four samples did show a significant peak shift, larger when seen on the fivefold axis and with a α value of the order −0.01, *i.e.* slightly larger than in the present sample.

The present results demonstrate that the diffraction pattern of the i-ScZn_7.33_ departs slightly but distinctly from the perfect icosahedral symmetry, unlike in the case of i-Sc–Zn–Mg for instance (de Boissieu *et al.*, 2005 ▸). We cannot say whether the structure is locked on a periodic AP or not, but from the value of the phason strain, the smallest periodic AP compatible with the present findings would have a cubic unit cell with a lattice parameter larger than 90 Å.

In the next step, we have investigated the Bragg peak broadening by measuring the longitudinal and transverse Bragg peak width for a selection of about 16 reflections lying in a broad *Q* 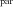 and *Q* 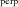 range along the twofold and fivefold axes. Each reflection was first centred and then measured with two different settings in transverse and longitudinal geometries. The FWHM of these reflections measured along the longitudinal and transverse directions were plotted as a function of *Q* 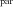 and *Q* 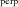 (see Fig. S4 in the supporting information). Whereas there is no visible trend for the evolution of the FWHM as a function of *Q* 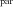, a clear linear dependence is observed when plotted as a function of *Q* 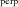. We observe, however, an anisotropic broadening, with a faster increase of the FWHM as a function of *Q* 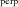 when measured in transverse geometry, *e.g.* the slope β of the linear relationship of the FWHM of the fivefold reflections as a function of *Q* 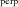 is equal to 3.8 × 10^−3^ and 6.7 × 10^−3^ for longitudinal and transverse directions, respectively. This is larger than what was found in the i-Al–Pd–Mn for which the value of β is equal to 2 × 10^−3^ and 4 × 10^−4^ for the as-grown and annealed samples, respectively (Létoublon *et al.*, 2001*b* ▸; Gastaldi *et al.*, 2003 ▸). In addition, when comparing as-cast and annealed i-ScZn_7.33_ samples, the β value for the longitudinal width for the fivefold reflections is found to be 4.3 × 10^−3^ and 3.8 × 10^−3^, respectively. This result indicates that the annealing indeed reduces the phason strain distribution in the sample.

The origin of this linear phason strain distribution is still an open question. It is known that dislocations in quasicrystals are accompanied by both a phonon and a phason strain distribution (Lubensky *et al.*, 1986 ▸; Socolar & Wright, 1987 ▸). It can thus be guessed that the present phason strain distribution is related to the presence of dislocations, but further experiments are required to validate this hypothesis. In the following we will treat the results within the icosahedral space group. This approach is justified by the very weak deviations from the ideal description, deviations that could only be evidenced because of the use of a high-resolution diffraction setup.

## 4. Atomic structure of i-ScZn_7.33_   

### 4.1. Synchrotron single-crystal X-ray diffraction data   

**Figure 4:**
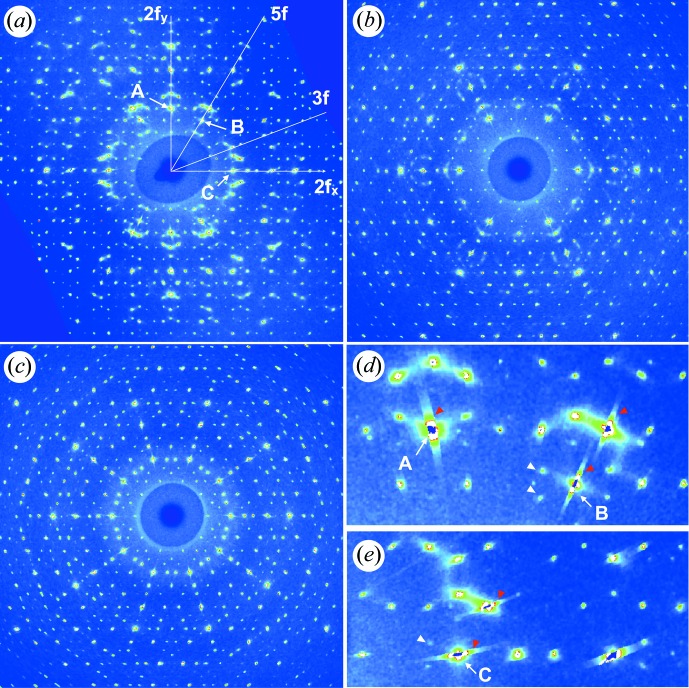
Reciprocal-space sections reconstructed from the diffraction data for the annealed i-ScZn_7.33_ sample, perpendicular to (*a)* two-, (*b*) three- and (*c*) fivefold axes. The lines on the twofold plane indicate two-, three- and fivefold axes. (*d*) and (*e*) are magnified pictures of (*a*) showing the diffuse scattering and peak shift, respectively. The reflections labeled by *A*, *B* and *C*, respectively are indexed as 20/32y, 18/29 and 20/32x reflections after Cahn *et al.* (1986 ▸). The white arrowheads indicate the peaks shifted from the ideal positions. The red arrowheads indicate streaks due to the saturation.

Fig. 4 ▸ shows the reciprocal-space sections of twofold, threefold and fivefold planes, reconstructed from the non-attenuated diffraction data for the annealed i-ScZn_7.33_ sample collected on the CRISTAL beamline. A few strong reflections are indicated using the N/M indexing scheme after Cahn *et al.* (1986 ▸). More than 290 000 Bragg peaks were observed, leading to 4778 unique reflections in a *Q*-range up to 16 r.l.u. The maximum *Q* 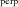 value necessary for the indexing was less than 3 r.l.u. On the twofold section, a large amount of diffuse scattering is present around strong Bragg reflections such as the 20/32 and 18/29 on the twofold section. As will be shown later, such strong diffuse scattering is due to long-wavelength phason fluctuations in the sample. In addition, we notice small displacements of some weak reflections from their ideal position, which can be explained by the presence of the linear phason strain in the sample as shown in the previous section.

### 4.2. Phase retrieval   

**Figure 5:**
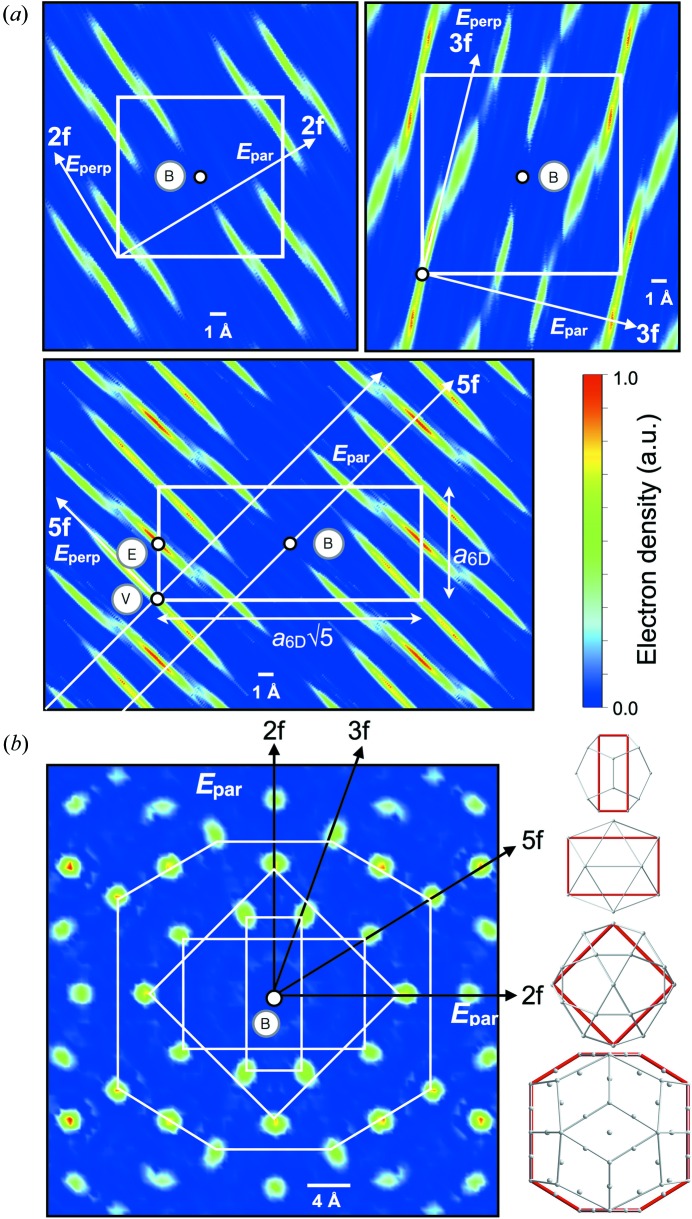
(*a*) Section of the six-dimensional election density distribution for i-ScZn_7.33_ cut by planes containing twofold, threefold and fivefold axes both in 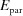 and 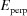. The high symmetrical positions of the six-dimensional lattice, (0,0,0,0,0,0), (1,1,1,1,1,1)/2 and (1,0,0,0,0,0)/2, are labeled by V, B and E, respectively. (*b*) Two-dimensional cut of six-dimensional electron density distribution for i-ScZn_7.33_ in the physical space 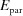 normal to a twofold axis (left), and an observed Tsai type RTH cluster (right).

Using the experimental data set, we carried out a structure analysis of the i-ScZn_7.33_. Firstly, the phase retrieval of the structure factors was carried out by the low-density elimination method (Shiono & Woolfson, 1992 ▸; Takakura *et al.*, 2001*b* ▸), which has already been applied successfully to several iQCs (Takakura *et al.*, 2001*b* ▸, 2007 ▸). This method was employed with 2921 unique reflections [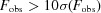] utilizing the *lodmac* software (Takakura *et al.*, 2001*b* ▸). In the next step, the six-dimensional electron density was obtained by Fourier synthesis of the structure amplitudes with the retrieved phases, utilizing the *qcmem* software (Yamamoto, 2008 ▸). Fig. 5 ▸(*a*) shows sections of the six-dimensional electron density distribution cut by planes containing two-, three- and fivefold axes both in 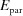 and 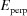. When compared with that found for the i-YbCd_5.7_ (Takakura *et al.*, 2007 ▸), the obtained electron density distribution of i-ScZn_7.33_ is quite similar, thus indicating that i-ScZn_7.33_ is a Tsai-type iQC. Indeed, from the three-dimensional electron density of the i-ScZn_7.33_ normal to a twofold axis (Fig. 5 ▸ *b*), an atomic arrangement of Tsai-type RTH cluster can be found (Fig. 5 ▸ *b*).

Although there is a large similarity between the density maps of i-ScZn_7.33_ and i-YbCd_5.7_, one of the main questions remaining is the explanation for the difference in their chemical compositions; there is in the i-ScZn_7.33_ QC a Sc (large atom) ‘deficiency’ which has to be explained. It has been proposed that Zn atoms occupy the two Yb or Sc sites inside the DFP (Tsai, 2013 ▸). The Sc contrast is not sufficient to extract this information from the electron density maps, thus it is necessary to refine the structural parameters against the intensity data in order to answer the question.

### 4.3. Structure refinement   

The structure refinement of i-ScZn_7.33_ QC was carried out based on the six-dimensional structure model of i-YbCd_5.7_ (Takakura *et al.*, 2007 ▸), by a least-squares method against 4057 unique reflections [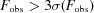], utilizing the *qcdiff* software (Yamamoto, 2008 ▸). As in the case of i-YbCd_5.7_, the ODs are decomposed according to the cluster ODs. This decomposition is however made on a smaller scale. This decomposition of the ODs in the asymmetric units then allows the introduction of a shift in 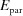 for each sub-OD, a parameter that is crucial in the refinement process and allows the description of the crystal chemistry of the iQC. Details of the six-dimensional structure model are described in the supporting information.

We started from a model in which the Yb icosahedron site is fully replaced by Sc, while the two Yb sites inside the DFP are substituted by Zn, which result in a composition of ScZn_7.6045_. The other Cd sites were fully replaced by Zn except for the disordered tetrahedron at the cluster center where 20 sites are occupied by four Zn atoms with an occupancy of 0.2. During the refinement process the Sc occupancy on the icosahedron site was fixed and the Sc/Zn ratio on the two Yb sites inside the DFP were refined together with other parameters such as the shift of ODs in 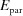, atomic displacement parameters (ADPs) in 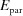 as well as an overall perpendicular (or phason) Debye–Waller (DW) factor, 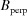. The last one corresponds, in a first approximation, to fluctuations of the OD along *E* 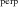.

The refinement converged with reliability (*R*) factor *R* = 0.1133 and *wR* = 0.0561 against 202 parameters (refinement 1). We found that one site inside the DFP (labeled 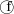 in Fig. S2 of the supporting information) is a mixed site with a Sc/Zn ratio of 0.145 (5)/0.86 (5), while the other site (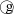 in Fig. S2) is fully occupied by Zn, thus resulting in the chemical composition of ScZn_7.38_, which is in agreement with that reported by Canfield *et al.* (2010 ▸). However, on the residual six-dimensional electron density map, a relatively high residual density peak was found at the position where the OD generates the Sc_12_ icosahedron shell (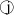 in Fig. S2) and is equal to 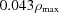. This indicates the presence of the Sc/Zn disorder on this site.

In a refinement with a further modified model where the Sc/Zn ratio on the icosahedron site was allowed to change, the refinement converged with a decreased *R*-factor with *R* = 0.1090 and *wR* = 0.0529 against 199 parameters (refinement 2). We observed a mixed occupancy on the icosahedron site with a relative occupancy of Sc/Zn = 0.74 (1)/0.26 (1). For the two sites inside the DFP one site (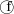 on Fig. S2) is fully occupied by Sc and the other (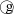 in Fig. S2) is a mixed site of Sc and Zn with a relative occupancy Sc/Zn = 0.48 (6)/0.52 (6). This model results in the chemical composition of ScZn_7.52_. Consequently, the residual density peak at the position discussed above decreases to 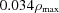. Plots of observed and calculated structure factors and the electron density maps are provided in the supporting information.

From the above refinements a possible Sc/Zn disorder on the icosahedron site results in a lower *R*-factor compared with the ordered model. Taking into account the level of complexity of the QC structure, it seems difficult to justify either of these solutions, and thus further studies in this direction are necessary. However, the results for the residual electron density distribution analysis support the scenario of a Sc/Zn disorder on the icosahedron shells. Below we present the structure description of i-ScZn_7.33_ obtained when a mixed Sc/Zn occupancy is assumed for the icosahedron shell (refinement 2).

**Figure 6:**
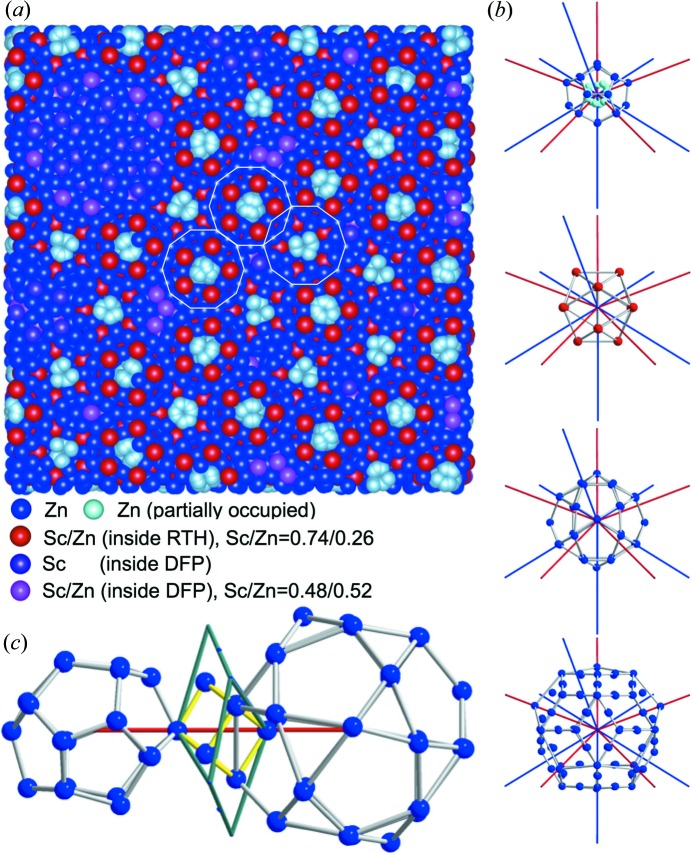
Resulting atomic structure of i-ScZn_7.33_. (*a*) An example of an 80 × 80 × 40 Å^3^ slab of the refined i-ScZn_7.33_ structure normal to a fivefold axis. White lines indicate RTH shells. (*b*) Tsai-type RTH cluster shells with the local configuration (12, 7, 5). The red and blue lines indicate *b*- and *c*-linkages, respectively. (*c*) Dodecahedron shell at (12, 7, 5) and icosidodecahedron shell in the neighbouring RTH cluster, linked by *c*-linkage. The green line indicates the overlapping part of the RTH shells, defining an oblate rhombohedraon. The yellow lines indicate the Zn_8_ cube.

Fig. 6 ▸(*a*) shows the projection of a slab with the size of 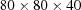
 Å^3^, normal to a fivefold axis, of the resulting three-dimensional atomic structure of i-ScZn_7.33_. The atoms are packed with a point density equal to 0.0636 Å^−3^. In the structure, the shortest interatomic distance, except for the innermost Zn_4_ tetrahedron, is about 2.08 Å, between the edge center site of the RTH shell and the Sc/Zn site inside the DFP (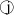 on Fig. S2). Yet, this distance does not occur frequently. The second one is about 2.27 Å, between the icosidodecahedron site and the edge center site on the RTH shell.

Whereas all the structural information is contained in the six-dimensional model in a very compact way, the resulting three-dimensional structure is best described in terms of the cluster local environment, *i.e.* the number of twofold and threefold cluster–cluster bonds and their configurations. In the 12-fold vertices of the three-dimensional AKN tiling the local environment of a RTH cluster is expressed by a notation (*Z*, 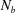, 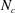) proposed by Henley (1986 ▸), where *Z* is the coordination number, while 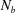 and 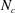 is the number of *b*- and *c*-linkages in *Z*, respectively. There are 18 local configurations in the i-YbCd_5.7_ model and their frequencies have been evaluated numerically, and the most frequent ones are (12, 7, 5) and (12, 6, 6), occurring with a probability of 24.87% and 24.40%, respectively (Takakura, 2008 ▸).

Figs. 6 ▸(*b*)–(*c*) show an RTH cluster of the refined structure of i-ScZn_7.33_, having the local configuration (12, 7, 5), together with the *b*- and *c*-linkages. Similar to what was observed in the i-YbCd_5.7_ case, large distortions are found on the Zn_20_ dodecahedron shells along the *c*-linkages. This distortion arises from the central Zn_4_ tetrahedron: indeed in the latest refinement for i-YbCd_5.7_, although the central Cd_4_ tetrahedron is modelled by a partially occupied icosahedron, after the refinement the atoms tend to be ‘grouped’ in a way that resemble a tetrahedron (Takakura, unpublished result).

This central Zn_4_ tetrahedron induces a distortion of the Zn_20_ dodecahedron in a similar way as what was observed in the low-temperature phase of the ScZn_6_ 1/1 cAP (Ishimasa *et al.*, 2007 ▸; Yamada *et al.*, 2013 ▸). As a result the Zn_20_ dodecahedron atoms lying on the *c*-linkages are ‘pushed’ away from the center leading to an enlargement of the triangles of the Zn_30_ icosidodecahedron. This then results in the distortion of the RTH shell. The distortion of the RTH shell is also reflected by the distortion of the neighboring RTH cluster, which is connected along the *c*-linkage, since two adjacent RTH clusters share a part of the atoms that belong to both the Zn_20_ dodecahedron and Zn_32_ icosidodecahedron (see Fig. 6 ▸ *c*). On the other hand, the distortion of the Sc_12_ icosahedron shell is relatively small and the shell shows almost perfect icosahedral symmetry, with the average Sc—Sc distance and its standard deviation being 5.19 and 0.11 Å. This distortion of the RTH cluster is very similar to that observed in the low-temperature phase of the 1/1 cAP (Ishimasa *et al.*, 2007 ▸; Yamada *et al.*, 2013 ▸), and is a direct consequence of the structure trying to be as compact as possible around the central Zn_4_ tetrahedron.

**Figure 7:**
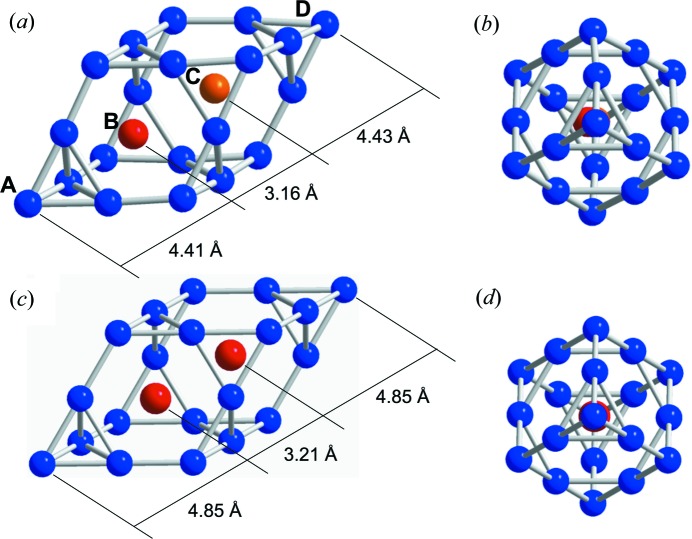
(*a*) A DFP located around the RTH cluster having the local configuration (12, 7, 5) in the refined i-ScZn_7.33_ structure. (*b*) A view along the longer body diagonal of (*a*). (*c*) A DFP in the ScZn_2_ Laves phase. (*d*) View along the longer body diagonal of (*c*). Red, blue and orange indicate Sc, Zn and Sc/Zn sites, respectively.

The chemical disorder that has been refined in the DFP is also inducing distortion of the Friauf polyhedron, since a large Sc atom is partially replaced by a smaller Zn atom. This is exemplified in Fig. 7 ▸, which shows a DFP around the RTH cluster having the local configuration (12, 7, 5) in the i-ScZn_7.33_ (Figs. 7 ▸ *a*–*b*) and that in the ScZn_2_ Laves phase (Liu *et al.*, 1996 ▸, Figs. 7 ▸ *c*–*d*). In the i-ScZn_7.33_ we find that the chemical disorder occurs for one of the two sites located along the longer body diagonal (site labelled C in Fig. 7 ▸ *a*): whereas, one site is fully occupied by Sc, the other site (C) is occupied by Sc or Zn. When the two sites inside the DFP are occupied by Sc, as in the case of the Laves phase (Fig. 7 ▸ *c*), the DFP is not distorted. On the other hand, when the C-site is occupied by Zn, this induces a distortion of the DFP with a shorter body diagonal, because the atomic radius of Zn is smaller than that of Sc. This interpretation is consistent with the determined length of the body diagonal which is equal to 12 Å in the i-ScZn_7.33_ quasicrystal to be compared with 12.9 Å in the Laves phase as shown Figs. 7 ▸(*a*)–(*c*).

We have thus achieved a refinement, leading to an atomic structure of the QC whose crystal chemistry is quite well understood. As for the 1/1 ScZn_6_ low-temperature phase, the close packing of the large Sc and small Zn atoms on one hand, and of the central Zn_4_ tetrahedron on the other hand, lead to a significant distortion of the different shells constituting the Tsai-type RTH cluster. This distortion is mainly driven by the threefold *c*-linkages. Moreover, the DFP is also significantly distorted, because of the replacement of Sc by Zn.

## 5. Phason diffuse scattering   

A significant diffuse scattering intensity is clearly visible in the diffraction pattern shown in Fig. 4 ▸; it is located near the Bragg peaks, and displays a shape elongation along directions parallel to the threefold axis. This anisotropic shape of the diffuse scattering cannot be explained by thermal diffuse scattering (TDS), but can be interpreted by the PDS in the framework of the hydrodynamic theory. Indeed, because of the icosahedral symmetry, the shape of TDS contribution must be the same for all Bragg reflections and has the form of an ellipsoid, elongated along the transverse directions.

Long-wavelength phason modes, which are fluctuations characteristic of the aperiodic state, also lead to diffuse scattering located near Bragg peaks. In the case of the iQC, and by neglecting the phonon–phason coupling term, the shape of the PDS is given by the two phason elastic constants 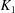, and 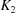. The PDS intensity, measured at the distance 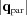 from a Bragg peak with a scattering vector **Q** having the parallel and perpendicular space components of 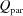 and 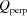, can be written as
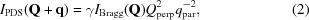
where the coefficient γ depends on the eigenvector and the eigenvalues of the hydrodynamic matrix C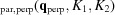. Here, the C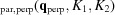 depend on 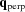 and the two phason elastic constants 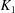 and 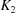. The ratio 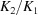, which varies in the range from −0.6 to 0.75, determines the shape of the PDS (Jarić & Nelson, 1988 ▸; Widom, 1991 ▸; Ishii, 1992 ▸; Rochal, 2001 ▸; Francoual, 2006 ▸; Francoual *et al.*, 2006 ▸). Because the PDS intensity is proportional to the 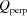 component of the Bragg peak under investigation, the PDS can be distinguished from the TDS.

**Figure 8:**
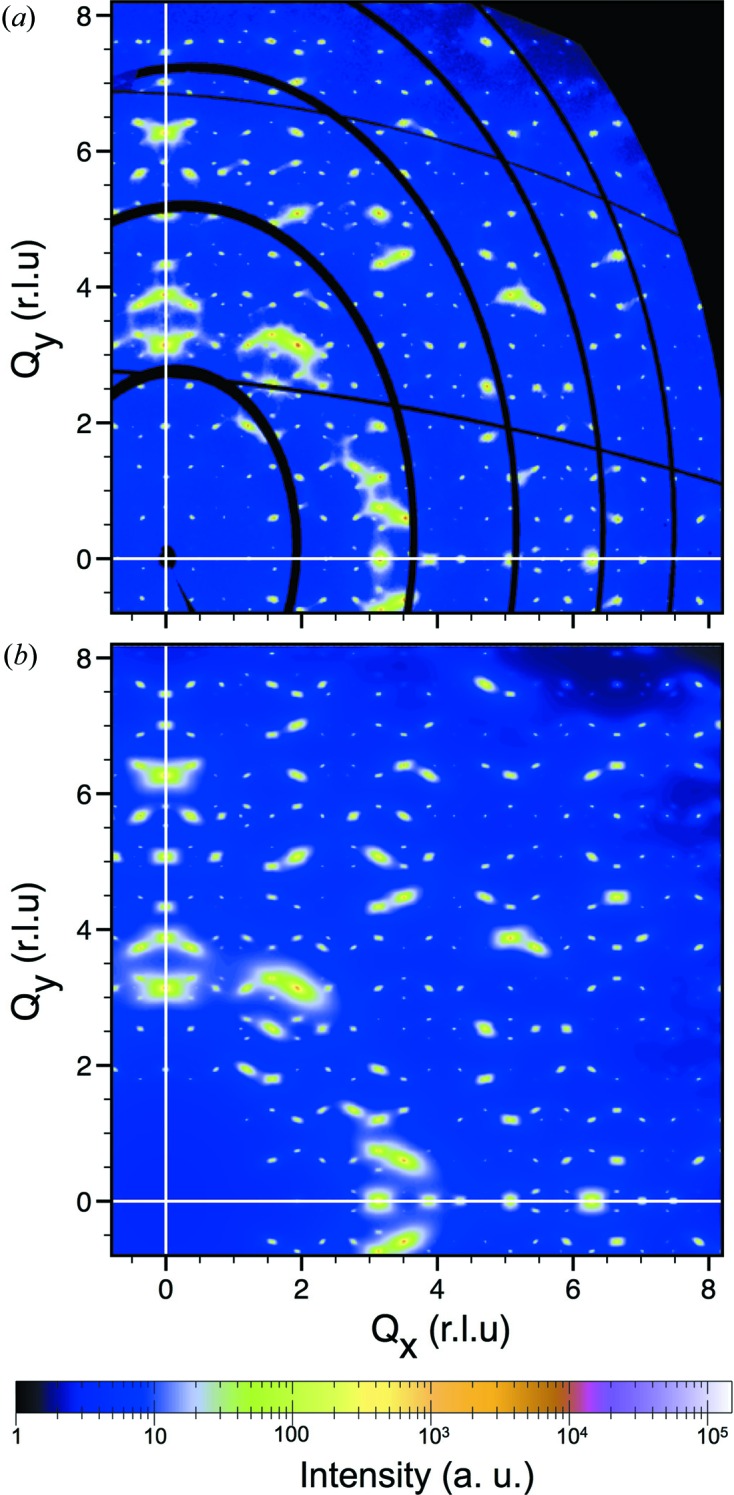
Comparison of experimental and simulated diffuse scattering on the twofold layer for i-ScZn_7.33_. (*a*) Diffuse scattering measured on the annealed i-ScZn_7.33_ sample. The diffraction data were collected at 300 K on ID23-1 at ESRF, using the Pilatus6M detector located at a distance of 200 mm from the sample. (*b*) Simulation of the same portion of reciprocal space. The simulation was carried out based on the elastic theory of iQC with the estimated elastic constants 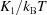 = 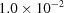 (Å^3^), 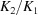 = −0.53 and 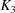 = 0.

Since the diffuse scattering measurement has been carried out on an absolute scale, the absolute value of the phason elastic constants can be determined. For this purpose we follow the same procedure as proposed by Francoual (2006 ▸) and have measured the diffuse intensity along different 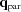 directions around different Bragg peaks (having different 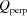 components). We found that the 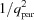 decay is valid in the 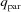 range from 0.05 to 0.5 Å^−1^ corresponding to a wavelength between 12 and 125 Å (see Fig. S9 in the supporting information). Measuring the quantity 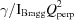 we have deduced the numerical values of the phason elastic constants which are found to be equal to 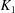/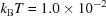
 Å^−3^, 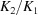 = −0.53. Using these values of the phason elastic constants, we have calculated the PDS intensity and have compared it to the experimental one. An excellent agreement between calculation and observation has been obtained as shown in Fig. 8 ▸: in particular the anisotropic shape of the PDS is perfectly reproduced around different Bragg peaks. Moreover, the diffuse scattering distribution was found to be similar in the annealed and as-cast samples in terms of shape anisotropy, as was also found for the i-Al–Pd–Mn phase (Létoublon *et al.*, 2001*a* ▸).

The 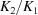 ratio is close to the limit of the first-order threefold instability limit which is predicted for a value of −0.6. Furthermore, it is very similar to what was shown in the i-Al–Pd–Mn QC phase (de Boissieu *et al.*, 1995 ▸; Boudard *et al.*, 1996 ▸; Létoublon *et al.*, 2001*a* ▸). In this case, it was found that the diffuse scattering increases as the temperature decreases, in agreement with a softening of the phason elastic constants. The PDS was thus interpreted as resulting from pre-transitional fluctuations with a breaking and subsequent lowering of the icosahedral symmetry, driven by a phason softening, which would be quenched in below 700 K for kinetic reasons. We can make the same hypothesis here, although this remains to be demonstrated by a high-temperature study.

### 5.1. Comparison between iScZn_7.33_ and i-Sc–Zn–Mg QCs   

**Figure 9:**
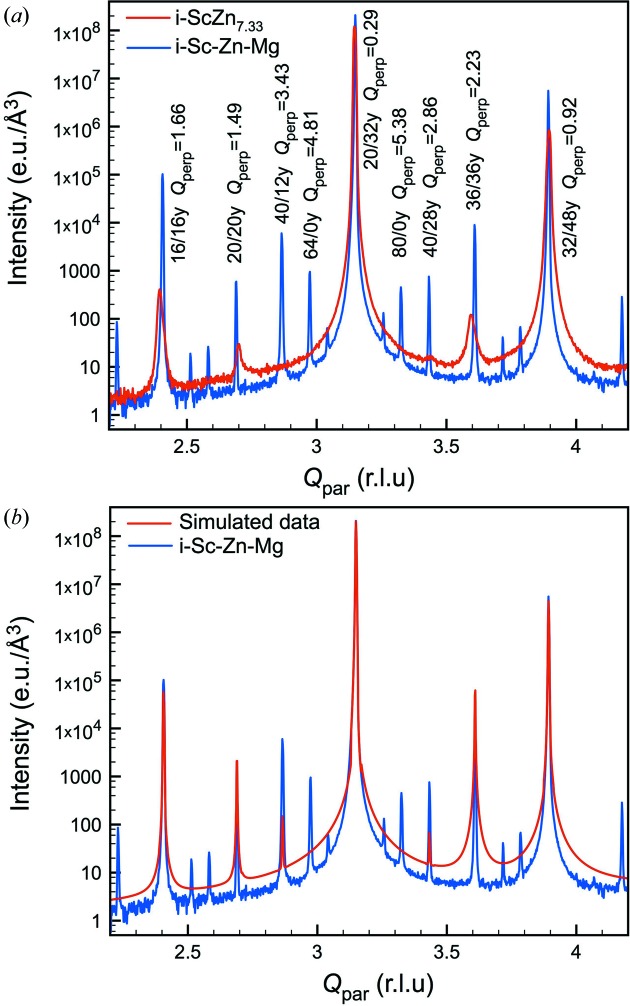
(*a*) Comparisons of the experimental diffuse scattering intensity distribution between the annealed i-ScZn_7.33_ and i-Sc–Zn–Mg samples as measured along a twofold axis. Bragg peaks are indexed by their N and M indices following the Cahn *et al.* scheme. (*b*) Comparison between the experimental diffuse intensity measured in the i-Sc–Zn–Mg sample, and a simulated diffraction pattern of the i-ScZn_7.33_ phase. The simulated diffraction pattern was obtained using the determined phason elastic constant and the phason DW factor, applied onto the experimental data of i-Sc–Zn–Mg. It reproduces well the observed one as shown in Fig. 9 ▸(*a*). The experimental diffuse intensity data for i-Sc–Zn–Mg is from de Boissieu *et al.* (2005 ▸).

It is interesting to compare the diffuse scattering intensity with that measured for the i-Sc–Zn–Mg phase on an absolute scale. As mentioned above, i-Sc–Zn–Mg is known to have the best quality among iQCs studied so far, *i.e.* low diffuse scattering, no deviation of the Bragg peaks from the icosahedral symmetry (de Boissieu *et al.*, 2005 ▸), and is considered to be isostructural to i-ScZn_7.33_. Fig. 9 ▸(*a*) compares the longitudinal scan of Bragg peaks on a twofold axis for the annealed i-ScZn_7.33_ and i-Sc–Zn–Mg QCs. There are two striking features:

(1) There is a much larger number of weak, high-*Q* 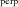, reflections in the i-Sc–Zn–Mg diffraction pattern compared with the i-ScZn_7.33_ one. Indeed the indexing of the i-Sc–Zn–Mg diffraction pattern requires *Q* 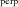 values up to 7 r.l.u. (de Boissieu *et al.*, 2005 ▸) to be compared to only 3 r.l.u. in the case of the i-ScZn_7.33_.

(2) There is a larger amount of diffuse scattering in i-ScZn_7.33_. The difference is larger around high-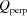 reflections as expected for a PDS contribution (see for instance the 32/48*y* twofold reflection).

These observations can be explained by taking into account the perpendicular DW factor, 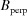. In fact, an increase in phason fluctuations will induce an increase of the PDS, but also an increase of the 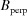 value. If we approximate the phason fluctuations by a Gaussian fluctuation of the OD with 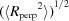 their mean square displacement, the factor 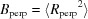 diminishes the structure factor of a given reflection as
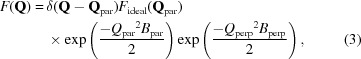
where 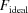 stands for the structure factor of the perfect structure, 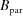 is the DW factor in 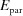, and *Q* 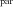 and *Q* 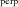, expressed in Å^−1^, are the parallel and perpendicular space components of the six-dimensional Bragg peak, respectively.

Since the phason contribution in the i-Sc–Zn–Mg phase is very weak, we can have an estimate of the 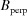 value in the i-ScZn_7.33_ phase by comparing the integrated intensity of the two phases. Although the two data sets were not collected under the same conditions (different incident energy) and one of the phases contains a weak scatterer (Mg), the logarithmic plot of the ratio of the measured structure factors of the two phases as a function of 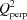 displays a clear trend, showing that the intensities of the i-ScZn_7.33_ phase are systematically smaller (see Fig. S10 in the supporting information). From this we can estimate that 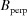 is of the order 0.76 Å^2^. It should be noted that the meaningful value is the ratio of the mean square displacement against the size of the OD because the reciprocal lattice constant in perpendicular space 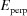 is in arbitrary units. The two ODs at the vertex and body-centred positions have a radius almost equal to that of a rhombic triacontahedron that generates the three-dimensional AKN tiling whose radius is equal to 8.12 Å (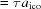) along the fivefold direction. 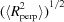 is approximately 10% of these ODs radii. In addition, this is approximately 30% of the radius of the OD that generates the cluster centres (radius equal to 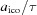). This will create a significant amount of disorder.

This is to be compared to the value obtained from the structure refinement. In the refinement 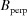 was found to be equal to 2.05 (5), which corresponds to 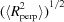 values of about 10% of the OD for the three-dimensional AKN tiling, respectively. Here, we note that, in the *qcdiff* software, the effect of the phason DW factor is expressed by 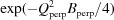, where 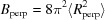, and the length scale in the perpendicular reciprocal space is set to 1.

The 10% fluctuation of the ODs is also fully consistent with the vanishing of high-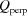 reflections as observed experimentally. For instance, 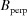 diminishes the intensity of a Bragg peak with 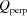 = 5 r.l.u. by a factor of 10^−6^. Fig. 9 ▸(*b*) shows the simulated diffraction pattern, obtained using the determined phason elastic constant and the phason DW factor, applied onto the experimental data of i-Sc–Zn–Mg from de Boissieu *et al.* (2005 ▸). When this simulated diffraction pattern is compared to that measured on i-Sc–Zn–Mg, it is clear that the high-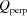 reflections, larger than 4.81 r.l.u. (such as 64/0y and 80/0y), vanish in the diffuse scattering. In addition, the resulting shape of the diffuse tails are very similar to the experimental ones of i-ScZn_7.33_ depicted in Fig. 9 ▸(*a*). This comparison thus confirms the validity of the determined phason DW factor, corresponding to fluctuations of the OD equal to 10% of their radius.

## 6. Discussion and summary   

The structure of the i-ScZn_7.33_ appears to be isostructural to the i-YbCd_5.7_ one and is described by a quasiperiodic packing of Tsai-type RTH clusters and DFPs, both resulting from the close-packing of a large (Sc) and a small (Zn) atom. The difference in their chemical compositions was found to result in chemical disorder that occurs on the large atom Sc sites, *i.e.* the icosahedron shell and one of the two sites located inside the DFP. The Sc/Zn occupancy on the icosahedron site is found to be equal to 0.76/0.24. Inside the DFP, one of the two sites remains fully occupied by Sc, whereas the other one has a mixed Sc/Zn occupancy equal to 0.48/0.52. Since the radius of Zn is smaller than that of Sc, the replacement of Sc by Zn induces a significant distortion of the DFP. Similar to what was observed in the i-YbCd_5.7_, we also observe significant distortions of the successive shells in the RTH cluster, mainly along *c*-linkages, that are induced by the central Zn_4_ tetrahedron.

Another important finding is the large amount of observed diffuse scattering, fully accounted for by phason fluctuations, and a ratio of the phason elastic constant 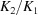 equal to −0.53, *i.e.* close to a threefold instability limit. The small value of 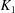 and the relatively large value of 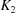, expressed in absolute scale, lead to a large average fluctuation of the ODs, of the order 10% of their overall diameter, which is fully consistent with the perpendicular DW factor obtained from the six-dimensional structure analysis. Both the derived phason elastic constants and perpendicular DW factors allow the quantitative reproduction of the observed diffraction pattern with a relatively small number of high-*Q* 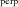 reflections and a significant amount of diffuse scattering. That the i-ScZn_7.33_ phase is close to a threefold instability limit is also consistent with the weak, but present linear phason strain.

Although, the underlying theory used for the diffuse scattering calculation is a continuum, hydrodynamic one, the absolute values we have derived will impose severe limits on possible atomistic models. In fact, we see three microscopic origins to explain those phason fluctuations:

(*a*) A cluster rearrangement (on the length scale of the cluster–cluster connection); such a cluster rearrangement will in fact require only a few atoms to be displaced.

(*b*) The replacement of Sc by Zn in the DFP. Although this is not proof, it is interesting to note that the DFP are oriented with their body diagonal parallel to a threefold axis.

(*c*) The degree of freedom of the central Zn_4_ tetrahedron inside the RTH cluster. Indeed, in the case of the ScZn_6_ 1/1 cAP but also in the QC, combining a quasi-elastic neutron scattering experiment with atomic scale simulation, it was demonstrated that the Zn_4_ tetrahedron behaves as a single molecule that reorients dynamically. This is accompanied by large distortions of the surrounding outer shells conferring an exceptional dynamical flexibility to the QC (Euchner *et al.*, 2012 ▸; Euchner *et al.*, 2013 ▸; Yamada *et al.*, 2013 ▸).

The large amount of observed diffuse scattering certainly points towards a significant entropy contribution from phason fluctuations. Atomic scale simulations and/or further experimental studies at various temperatures are required in order to decipher their microscopic origin. Such studies are also of importance to understand whether the proximity of the threefold phason type instability is related or not to a pretransitional state as was observed in i-AlPdMn (Boudard *et al.*, 1996 ▸).

On the other hand, it is interesting to look at the possible energy contribution to the stabilization mechanisms for the i-ScZn_7.33_ phase. In this close-packed structure, there are two main parameters to the energetics, *i.e.* valence electron concentration (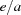) and atomic size factor (δ), which are related to a Hume–Rothery like stabilization, although in this case the stabilization is more to be related to the *sp*–*d* hybridization (Ishii & Fujiwara, 2001 ▸, 2002 ▸). The value of δ is defined by 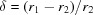, where 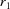 and 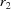 are atomic radii of the first (large) and second (small) elements, respectively. For the i-ScZn_7.33_, the 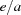 and δ values are equal to 2.12 and 0.177, respectively. When compared with that for the i-YbCd_5.7_ and isostructural i-Ca_15_Cd_85_ iQCs, we see that these two parameters for the i-ScZn_7.33_ deviate from that for i-YbCd_5.7_ and i-Ca_15_Cd_85_. For i-YbCd_5.7_ and i-Ca_15_Cd_85_ the values of 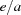 are 2.0, while the values of δ are equal to 0.237 and 0.259, respectively. On the other hand, recently discovered i-*R*-Cd (*R* = Y, Gd–Tm; Goldman *et al.*, 2013 ▸) have similar values of 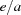 and δ to that for i-ScZn_7.33_. Firstly, the values of 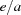 for i-*R*-Cd (*R* = Y, Gd–Tm) are close to 2.12. This is because the chemical compositions of i-*R*-Cd (*R* = Y, Gd–Tm) are very close to that of i-ScZn_7.33_ and the first elements in these phases are trivalent. Secondly, the δ values of i-*R*-Cd (*R* = Y, Gd–Tm) are between 0.149 and 0.106, and close to that of i-ScZn_7.33_. Therefore, these binary Tsai-type iQC phases can be divided into two groups in terms of 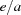, atomic size factor and valence of the first element; the first group includes i-YbCd_5.7_ and i-Ca_15_Cd_85_, and the second one i-ScZn_7.33_ and i-*R*-Cd (*R* = Y, Gd–Tm).

It is known that the 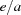 ratio is the dominant factor in the formation of stable iQCs and the iQC phases having the same structure type have a common 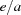 ratio (Tsai, 2004 ▸). Indeed, a recent structure analysis for i-*R*-Cd (*R* = Gd, Dy and Tm) revealed that a similar chemical disorder also exists, hence they may be grouped in the same structural class (Yamada *et al.*, 2016 ▸). The i-ScZn_7.33_ and i-*R*-Cd (*R* = Y, Gd–Tm) would thus form a new type of binary quasicrystalline structures, characterized by the same 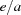 ratio, the same long-range quasiperiodic order and chemical disorder together with a similar phason diffuse scattering. One might thus expect for this quasicrystal family a unique energetic and entropic contribution to the total free energy and a similar underlying stabilizing mechanisms.

## 7. Related literature   

References cited in the supporting information include: de Boissieu (2008 ▸, 2012 ▸); de Boissieu *et al.* (1991 ▸, 2007 ▸); Boudard *et al.* (1992 ▸); Cahn *et al.* (1986 ▸); Cornier-Quiquandon *et al.* (1991 ▸); Elser (1986 ▸); Gratias *et al.* (2000 ▸); Henley (1986 ▸); Horn *et al.* (1986 ▸); Ishii (1989 ▸, 1992 ▸); Janssen (1986 ▸; 1991 ▸); Janssen *et al.* (2007 ▸); Jarić & Nelson (1988 ▸); Kramer & Neri (1984 ▸); Lubensky (1988 ▸); Lubensky *et al.* (1986 ▸); Quiquandon & Gratias (2006 ▸); Socolar & Wright (1987 ▸); Takakura *et al.* (2007 ▸); Widom (1991 ▸); Yamamoto (1992 ▸, 1996 ▸, 2008 ▸); Yamamoto *et al.* (2003 ▸).

## Supplementary Material

Crystal structure: contains datablock(s) ico-Sc-Zn. DOI: 10.1107/S2052252516007041/gq5006sup1.cif

Structure factors: contains datablock(s) ico-Sc-Zn. DOI: 10.1107/S2052252516007041/gq5006ico-Sc-Znsup2.hkl

Intensity data collection details. DOI: 10.1107/S2052252516007041/gq5006sup3.txt

Supporting figures and tables. DOI: 10.1107/S2052252516007041/gq5006sup4.pdf

CCDC reference: 1476647
