# Supplementary material for: Atomic structure and phason modes of the Sc–Zn icosahedral quasicrystal
Source: IUCrJ. 2016 Jun 14;3(Pt 4):247–58. doi: 10.1107/S2052252516007041 (PMC4937780; doi:10.1107/S2052252516007041)
Supplement: Supplementary file 4 [file m-03-00247-sup4.pdf]

IUCrJ

Volume 72 (2016)

Supporting information for article:

Atomic structure and phason modes of the Sc–Zn icosahedral quasicrystal

Tsunetomo Yamada, Hiroyuki Takakura, Holger Euchner, Cesar Pay Gómez, Alexei Bosak, Pierre Fertey and Marc de Boissieu

S1. Phason modes of quasicrystals

Here we give a rapid overview of 'phasons' in quasicrystals (QCs), since the term 'phason' has been used with quite different meanings in the field of QCs. This is best understood in the superspace formalism of aperiodic crystals schematically depicted in Fig. S1. The simplest case of a quasiperiodic structure is a one-dimensional (D) model such as the Fibonacci chain. In this case the structure is obtained from a 2-D periodic structure as exemplified in Fig. S1(a). The Fibonacci chain is formed from two different tiles, named *L* and *S*, where *L* and *S* are the long and short segments.

A first widely used meaning of the term phason, concerns the so-called long wavelength phason fluctuations, which are excitations characteristic of the aperiodic long-range order in aperiodic crystals, i.e. incommensurately modulated structures, incommensurate composite crystals and QCs (see (de Boissieu *et al.*, 2008) and (Janssen *et al.*, 2007) for an introduction). In the superspace description of aperiodic crystals, and within the hydrodynamic theory, phason modes are a consequence of the free energy invariance of the aperiodic structure with respect to a translation of the cut space E_{par} along the perpendicular space, E_{perp}. Indeed two aperiodic structures obtained from the section of the same superspace structure, but with a different translation of the cut space are in general not identical. However they are indistinguishable because they have the same free energy and produce the identical diffraction pattern. This free energy invariance with respect to a perpendicular space translation is sometimes called 'phason degree of freedom' which is at the basis of the prediction of long wavelength phason modes. In the long wavelength limit, the theory predicts for all aperiodic crystals that phason modes are diffusive like excitations, i.e. unlike phonons they are not propagative. For QCs a generalized theory of elasticity has been derived, with five elastic constants in the case of icosahedral (i) phases: the two Lamé coefficients (λ and μ), the phason elastic constants (K_1 and K_2) and the phonon-phason coupling term K_3 (Lubensky *et al.* 1988).

In the same way that phonons give rise to thermal diffuse scattering (TDS) on the diffraction pattern, phason fluctuations give rise to phason diffuse scattering (PDS). The PDS can be computed within the generalized elasticity theory (Jaric & Nelson, 1988; Widom, 1991; Ishii, 1992). When the phonon-phason coupling term can be neglected, the diffuse scattering intensity at a distance $|\mathbf{q}|$ from a Bragg reflection with a reciprocal vector $\mathbf{Q}_{\mathrm{par}}$ is expressed as

$$I_{\mathrm{diffuse}} = I(\mathbf{Q}_{\mathbf{par}} + \mathbf{q}) = I_{\mathrm{TDS}} + I_{\mathrm{PDS}}\ , \quad (1)$$

where the phason part depends on $\mathbf{Q}_{\mathrm{perp}}$, the two phason elastic constants K_1 and K_2, and the PDS decays as $1/q^2$ (see for details ref (de Boissieu, 2007)). Although the shape of the TDS is observed around Bragg peaks is identical (isotropic in the case of icosahedral phase), the PDS intensity distribution varies in Bragg peak to Bragg peak and their shapes depend on the ratio K_2/K_1 and the direction of $\mathbf{Q}_{\mathrm{perp}}$. The PDS thus is distinguishable from the ordinary TDS.

Because the hydrodynamic theory is a continuum theory it does not provide a view on a microscopic mechanism on the phason fluctuation. However at the atomic level, the fluctuation of the cut space along E_{perp} leads to atomic re-arrangements. In case of a 1-D QC, the Fibonacci chain, this corresponds to a *LS* to *SL* exchange as shown in Fig. S1(b). These tile reorganizations have been called 'phason flip', in the QC jargon.

A second widely used meaning of the term phason concerns the so-called phason strain, shown in Figure S1c. When the slope of the cut space changes, which is equivalent to a phason strain, this produces systematic atomic re-arrangements, resulting in a breaking of the icosahedral symmetry. On the corresponding diffraction pattern, Bragg peaks shift away from their ideal positions (Socolar *et al.*, 1987). The peak shift ($\Delta\mathbf{Q}_{\text{par}}$) can be expressed as a product between the so-called phason matrix **M** and the perpendicular reciprocal vector $\mathbf{Q}_{\text{perp}}$ of the Bragg reflection. The phason matrix **M** depends on the symmetry of the system that is a subgroup of the icosahedral symmetry, such as $m\bar{3}$, $\bar{3}m$ and $\bar{5}m$. In general the resulting structure remains quasiperiodic. For particular values of the phason matrix **M** a periodic structure (periodic approximant) is obtained. (Ishii, 1989; Janssen, 1991). When there is a random distribution of phason strain in the sample, the Bragg peaks are no longer delta functions: they have a finite width that scales linearly with $Q_{perp} = |\mathbf{Q}_{\text{perp}}|$ (Horn, 1986; Lubensky, 1986).

Phason diffuse scattering has been observed for all QC phases to date (see (de Boissieu, 2012) for a recent review). In addition Bragg peak broadening due to the distribution of phason strain is also present. Bragg peak shifts due to linear phason strain are often observed in transmission electron microscopy studies and also in some X-ray diffraction studies. These phenomena are crucial to evaluate the quasicrystal 'quality' i.e. the degree of perfection of the quasiperiodic long-range order of the sample under investigation.

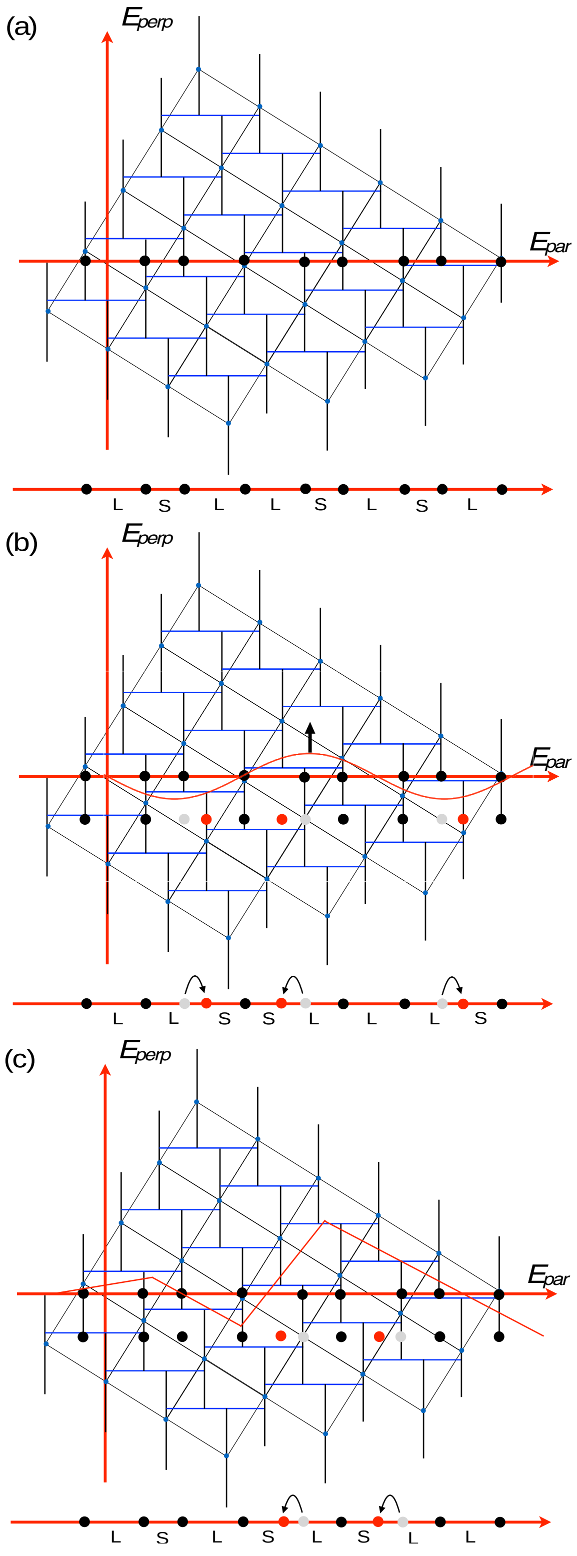

Figure S1 2-D representation of a phason mode in a 1-D quasicrystal. (a) a distribution of a 1-D quasicrystal, (b) a single component of the long wavelength phason fluctuations (c) and liner phason strain (c). Some points have 'jump' to a nearly equivalent site transforming a SL into LS sequence (gray and red colors). In the case of the phason strain distribution the amplitude of the fluctuations diverges at infinity, destroying the long-range order: in that case a Bragg peak broadening occurs. For the case of the phason fluctuations they are bounded in the perpendicular direction.

S2. Definition of the 6-D vectors for $ScZn_{7.33}$ icosahedral quasicrystal

There are several conventions for defining the basis vectors for the description of iQCs and therefore, there is no conventional indexing scheme. In the paper we used a definition of the orthonormal set of 6-D vectors proposed by Cahn *et al.* (1986) for indexing the diffraction pattern of the i-$ScZn_{7.33}$ QC. On the other hand, one proposed by Yamamoto (1996) was used for the structure analysis, because we used the computer package QUASI07_08 (Yamamoto, 2008) uses this setting. The correspondence between the six indices by Cahn *et al.* (1986) , n_1, …, n_6, and that by Yamamoto (1996), h_1, …, h_6, can be written as following;

$$\begin{pmatrix} h_1 \\ h_2 \\ h_3 \\ h_4 \\ h_5 \\ h_6 \end{pmatrix} = \begin{pmatrix} 1 & 0 & 0 & 0 & 0 & 0 \\ 0 & 1 & 0 & 0 & 0 & 0 \\ 0 & 0 & 0 & 0 & 0 & 1 \\ 0 & 0 & 0 & 0 & 1 & 0 \\ 0 & 0 & 1 & 0 & 0 & 0 \\ 0 & 0 & 0 & \bar{1} & 0 & 0 \end{pmatrix} \begin{pmatrix} n_1 \\ n_2 \\ n_3 \\ n_4 \\ n_5 \\ n_6 \end{pmatrix}. \quad (2)$$

The 6-D reciprocal lattice vector $\mathbf{Q}$ has two components and is denoted as $\mathbf{Q} = (\mathbf{Q}_{\text{par}}, \mathbf{Q}_{\text{perp}})$. In the indexing scheme by Cahn *et al.* (1986), the $\mathbf{Q}$ is defined by using a set of orthonormal 6-D vectors $\boldsymbol{n}_j$ $(j = 1, \dots, 6)$ which span the 6-D cubic lattice with a 6-D lattice constant a_{6D}. The 3-D reciprocal lattice vector $\mathbf{Q}_{\text{par}}$, which are employed for indexing Bragg peaks of icosahedral quasicrystals, are considered as the 3-D parallel space components of the corresponding 6-D reciprocal lattice vectors onto 3-D parallel space, E_{par}, as following;

$$\mathbf{Q}_{\mathbf{par}} = \begin{pmatrix} Q_{\text{par},1} \\ Q_{\text{par},2} \\ Q_{\text{par},3} \end{pmatrix} = c * \begin{pmatrix} 1 & \tau & 0 & -1 & \tau & 0 \\ \tau & 0 & 1 & \tau & 0 & -1 \\ 0 & 1 & \tau & 0 & -1 & \tau \end{pmatrix} \begin{pmatrix} n_1 \\ n_2 \\ n_3 \\ n_4 \\ n_5 \\ n_6 \end{pmatrix}, \quad (3)$$

where τ is the golden mean equals to $\frac{\sqrt{5}+1}{2}$ and c is a scale factor equal to $\frac{2\pi}{a_{6\text{D}}\sqrt{2(2+\tau)}}$.

Along the same way, the 3-D perpendicular space components of the corresponding 6-D reciprocal lattice vectors onto 3-D perpendicular space, E_{perp}, as following;

$$\mathbf{Q}_{\text{perp}} = \begin{pmatrix} Q_{\text{perp},1} \\ Q_{\text{perp},2} \\ Q_{\text{perp},3} \end{pmatrix} = c * \begin{pmatrix} -\tau & 1 & 0 & \tau & 1 & 0 \\ 1 & 0 & -\tau & 1 & 0 & \tau \\ 0 & -\tau & 1 & 0 & \tau & 1 \end{pmatrix} \begin{pmatrix} n_1 \\ n_2 \\ n_3 \\ n_4 \\ n_5 \\ n_6 \end{pmatrix}. \quad (4)$$

In the Yamamoto's scheme the lengths of $\mathbf{Q}_{\text{par}}$ and $\mathbf{Q}_{\text{perp}}$ are defined respectively by a^* and $a^{*\prime}$, which correspond to the reciprocal lattice constants in each sub-space. Conventionally, $a^{*\prime}$ is set to be the same length to a^* with the result that the 6-D reciprocal lattice is a 6-D cubic lattice. The lengths of $\mathbf{Q}_{\text{par}}$ and $\mathbf{Q}_{\text{perp}}$ are defined respectively by a and a', which correspond to the (direct) lattice

constants in each sub-space. In the paper, a is denoted as $a_{\rm ico}$, which is an icosahedral lattice constant equals to $a_{\rm 6D}/\sqrt{2}$, because the length corresponds to that of icosahedral quasilattice, which is obtained by projecting a subset of the 6-D cubic lattice onto 3-D parallel space. In this paper the structure refinement of the i-$ScZn_{7.33}$ QC was performed based on the 6-D structure model of the i-$YbCd_{5.7}$ QC (Takakura *et al.* 2007) whose occupation domains (ODs) are defined by using the setting of $a' = 1$. The position of each OD in the 6-D unit cell is specified by a fractional coordinate as in ordinary crystals. The detail of the 6-D structure model is described in next section.

S3. 6-D model of i-$YbCd_{5.7}$ QC

In the superspace description of iQCs their atomic arrangement in the parallel space, $E_{\rm par}$, is obtained as a result of an irrational cut of corresponding 6-D periodic structure. The 6-D periodic structure is described as a decorated 6-D lattice with 3-D objects, called occupation domains (ODs) or atomic surfaces, lying in the perpendicular space, $E_{\rm perp}$.

A simple example of iQC is 3-D Amman-Kramer-Neri (AKN) tilling (Kramer & Neri, 1984). The OD that generates the vertices of this 3-D AKN tiling is obtained as a projection of the 6-D unit cell, which has the shape of triacontahedron with an edge length of unity, onto $E_{\rm perp}$. The overall shape of this OD is shown in Fig. S2(a). This OD, a measure of the size of any ODs, can be defined using a set of three vectors specifying its asymmetric part. The first and the second vectors point respectively a five-fold and a three-fold vertices of the triacontahedron. The last one oriented in a two-fold direction points the center of a rhombus face. They are represented as the perpendicular space components of the corresponding 6-D vectors, $(1,1,-1,1,-1,-1)/2$, $(-1,1,-1,1,-1,-1)/2$ and $(0,1,-1,1,0,-1)/2$, respectively. Here the definition of the orthonormal set of 6-D vectors proposed by Yamamoto (1992) is used. The shape of the whole OD is obtained from the asymmetric part by the symmetry operations of the icosahedral group. Accordingly, the diameters of the OD along five-, three- and two-fold directions are 2τ, $(2+2\tau)/\sqrt{2+\tau}$ and $2\sqrt{3}\tau/\sqrt{(2+\tau)}$, respectively. The volume is $20(3-\tau)^{-3/2}$, which results in the point density:

$$\frac{5}{2}\left(\frac{\tau}{a\sqrt{2+\tau}}\right)^3. \qquad (5)$$

These values are equivalent to those found in a literature (Elser, 1986).

The 6-D structure model for the i-$YbCd_{5.7}$ QC (Takakura *et al.* 2007) is described based on so-called twelve-fold sphere-packing sites consisting of *b*- and *c*-linkages exclusively (Henley *et al.* 1986). They are the subset vertices of the 3-D Amman-Kramer-Neri (AKN) tilling. Fig. S2(b) shows the overall shape of the OD that generates the twelve-fold packing sites. This OD is obtained by modifying τ^{-2} times smaller triacontahedral OD in Fig. S2(a). In the 6-D model of the i-$YbCd_{5.7}$ QC this OD is configured to express the characteristic Tsai-type RTH clusters (Takakura *et al.* 2007).

Fig. S2(c) shows the entire ODs, which correspond to the densities observed at the positions V, E and B in Fig. 5 of the paper for the 6-D model of the i-$YbCd_{5.7}$ quasicrystal. The ODs at V and B are obtained by placing the archetype ODs suitably, so as to generate the atoms located at the vertices of the polyhedral shells of the RTH cluster shown in Fig. S2(e). The OD at E generates the atoms located at the mid-edges of a RTH and the DFP shown in Fig. S2(f). Its dimpled shape is due to the shape of the archetype ODs as a castingmold. No unreasonably short atomic distances appear in the physical space atomic structure except for the disordered tetrahedral sites located on the inner most shell of the RTH cluster. The structure refinements were carried out using the asymmetric parts of the ODs as shown in Fig. S2(d) by taking into account different local environments for the atomic sites.

The atomic structure in E_{par} can be described with two building blocks, namely, the RTH cluster and DFP, as shown in Fig. S2(e) and (f), in the case of Tsai-type icosahedral quasicrystal. The RTH cluster consists of five atomic shells: a central Cd_4 tetrahedron, a Cd_{20} dodecahedron, a Yb_{12} icosahedron, a Cd_{32} icosidodecahedron and a Cd_{92} RTH shells. The labels ⓐ − ⓚ in Fig. S2 distinguish the atomic sites with different local environments and also indicate the correspondence between the segmented ODs and the atomic sites on the RTH cluster and DFP. Two neighboring RTH clusters share a rhombus face with the distance *b* along the two-fold direction (*b*-linkage). Accordingly, the atomic sites labeled ⓒ and ⓓ in Fig. S3(e) have different local environments from the rest of the atomic sites on the RTH shell. A similar, but slightly more complicated, situation occurs when two RTH clusters interpenetrate, by sharing an oblate rhombohedron (OR), with the distance *c* along the three-fold direction (*c*-linkage). In this case, the atomic sites labeled ⓔ are distinguished by different local environments. The atomic site (ⓑ) on the vertex of the shorter body diagonal of the OR is associated with both the RTH shell of one cluster and the second dodecahedron shell of the adjoining cluster. The DFPs fills the interstitial voids of the RTH cluster network, and two Yb atoms (ⓕ and ⓖ) are located on the longer body diagonal of DFP.

Similarly to periodic crystal, there is some arbitrariness in defining the origin of the 6-D unit cell. In particular the origin can be shifted by the vector (1,1,1,1,1,1)/2. Applying this shift to the current 6-D model of i-$YbCd_{5.7}$ QC the OD that generates clusters centres is found at the vertex position, (0,0,0,0,0,0). Fig. S3 displays the two-, three- and five-fold sections of the 6-D electron density as shown in Fig. 5 but with the new origin shifted by the vector (1,1,1,1,1,1)/2. The position of the ODs are now at (0,0,0,0,0,0), (1,1,1,1,1,1)/2 and (0,1,1,1,1,1)/2 labelled by V, B and E, respectively. This choice of origin is similar to what had been done for the structure determinations (Janssen *et al.* 2007) of the Mackay-type QCs such as i-Al-Pd-Mn and i-Al-Cu-Fe (Cornier-Quiquandon *et al.*, 1991; Boudard *et al.*, 1992; Gratias *et al,*. 2000; Yamamoto *et al.*, 2003; Quiquandon & Gratias, 2006), and the Bergman-type QCs such as i-Al-Li-Cu (de Boissieu *et al.*, 1991; Yamamoto, 1992) so that the cluster generation can be directly compared. Indeed, the i-$YbCd_{5.7}$ QC contains 6-D 'ingredients' that are seen

in both the 6-D structure model for the Mackay and Bergman-type QCs. On the Fig. S3 one can see that for instance the Sc_{12} icosahedron is generated by the OD at V in a way similar for the larger icosahedron in Mackay-type iQC. On the other hand the Zn_{20} dodecahedron is generated by the OD at B similarly to the dodecahedron in Bergman-type iQC. The Zn_{32} icosidodecahedon is generated by the OD at V as for the icosidodecahedron in Mackay-type iQC. Finally, the large RTH is generated by the OD at B, and the small OD at E, now in the position (0,1,1,1,1,1)/2, generates a mid-edge decoration of the large RTH, that is characteristic to the Tsai-type iQC.

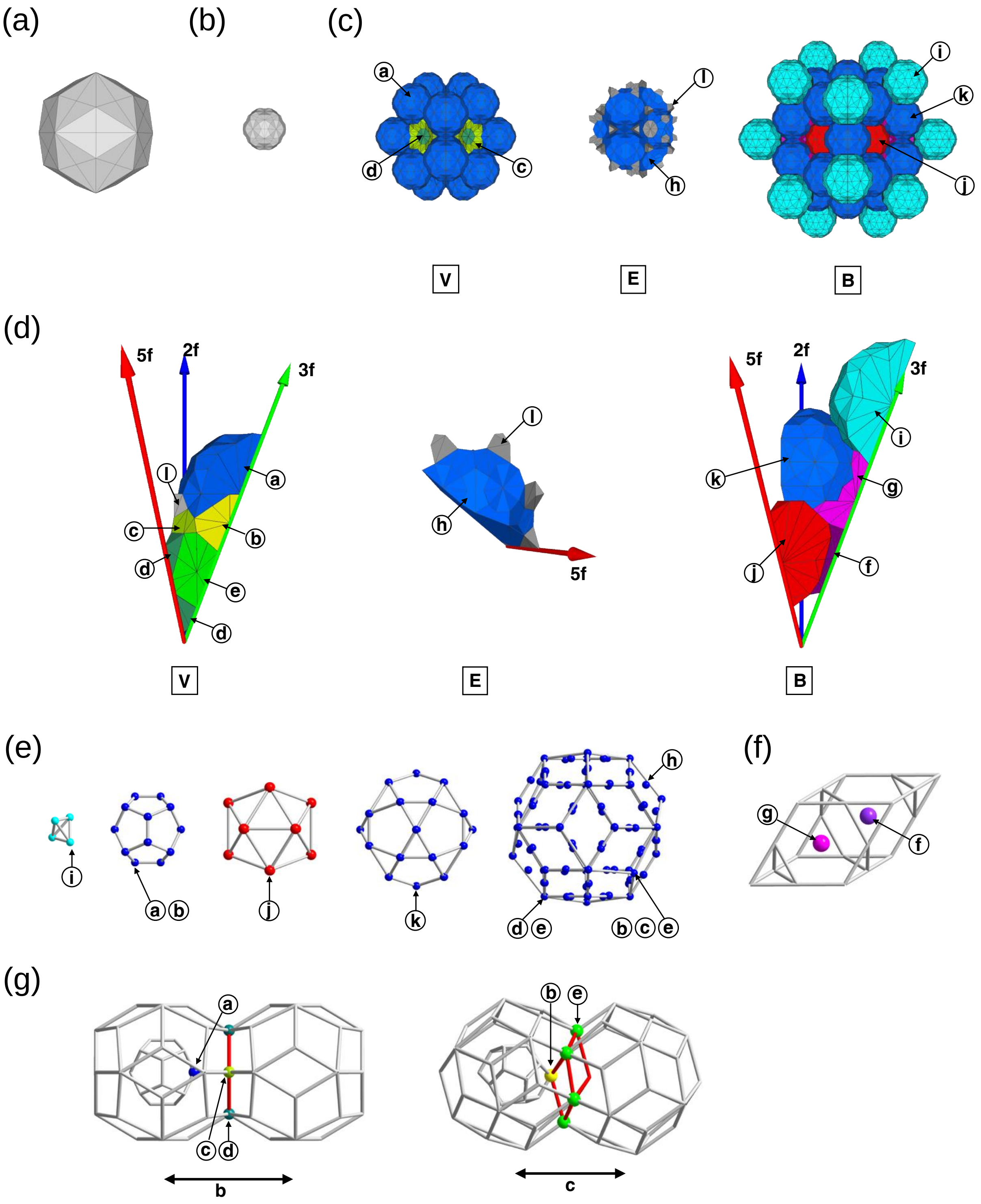

Figure S2 (a) OD for the vertices of a 3-D Amman-Kramer-Neri (AKN) tilling. (b) OD for the twelve-fold packing sites. (c) Overall ODs and (d) their asymmetric parts for the 6-D structure model for i-$YbCd_{5.7}$ QC (Takakura *et al.*, 2007). The ODs in (c, d) are located at the high symmetrical positions of the 6-D lattice, (0,0,0,0,0,0), (1,1,1,1,1,1)/2 and (1,0,0,0,0,0)/2 labeled by V, B and E, respectively. (e) Tsai-type rhombic triacontahedron (RTH) cluster and (f) double Friauf polyhedron (DFP). (g) *b*- and *c*-linkages of the RTH shells. The labels ⓐ - ⓚ distinguish the atomic sites with

different local environments and also indicate the correspondence relation between the segmented ODs and the atomic sites on the RTH cluster and DFP.

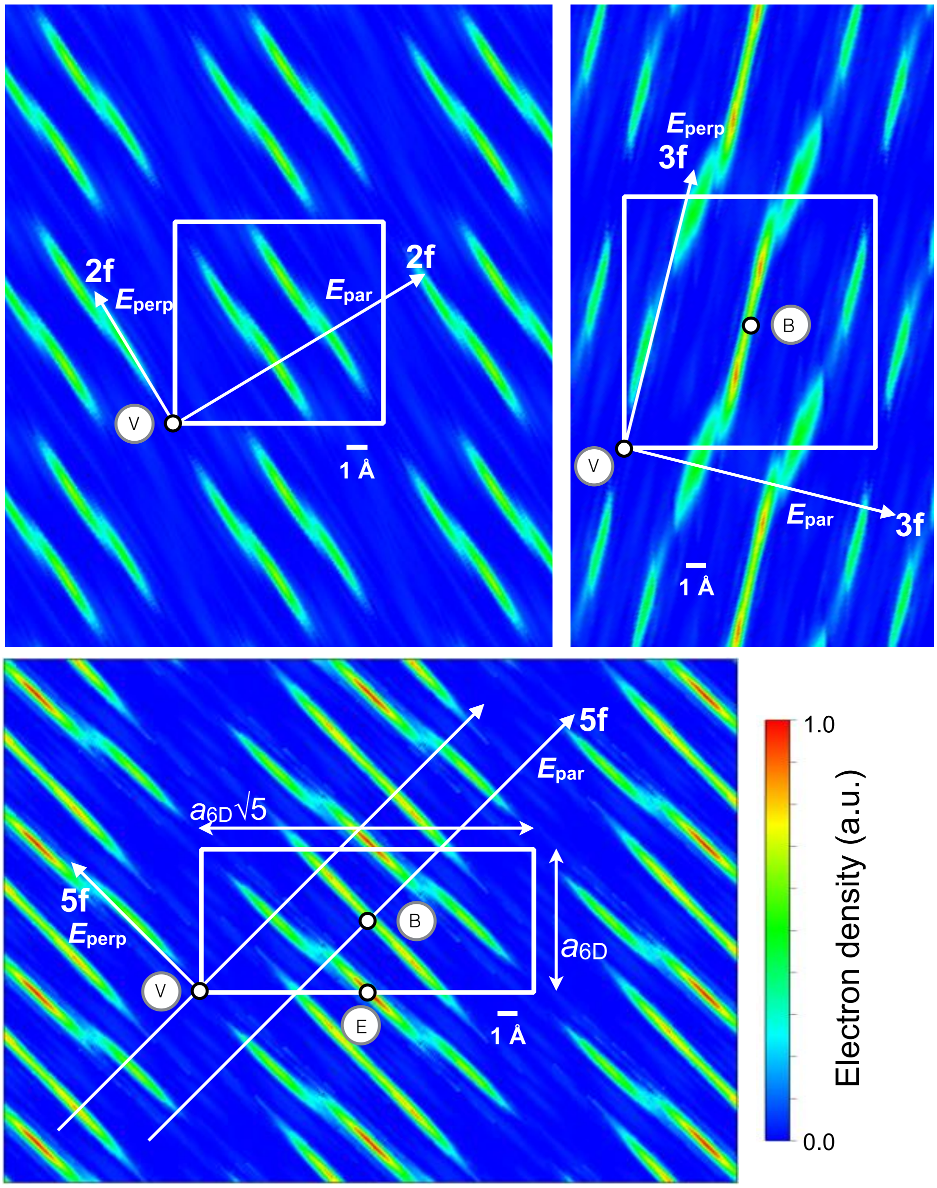

Figure S3 Same sections of the 6-D election density distribution for i-$ScZn_{7.33}$ shown in Fig. 5 but with the new origin shifted by the vector (1,1,1,1,1,1)/2. The high symmetrical positions of the 6-D lattice, (0,0,0,0,0,0), (1,1,1,1,1,1)/2 and (0,1,1,1,1,1)/2, are labelled by V, B and E, respectively.

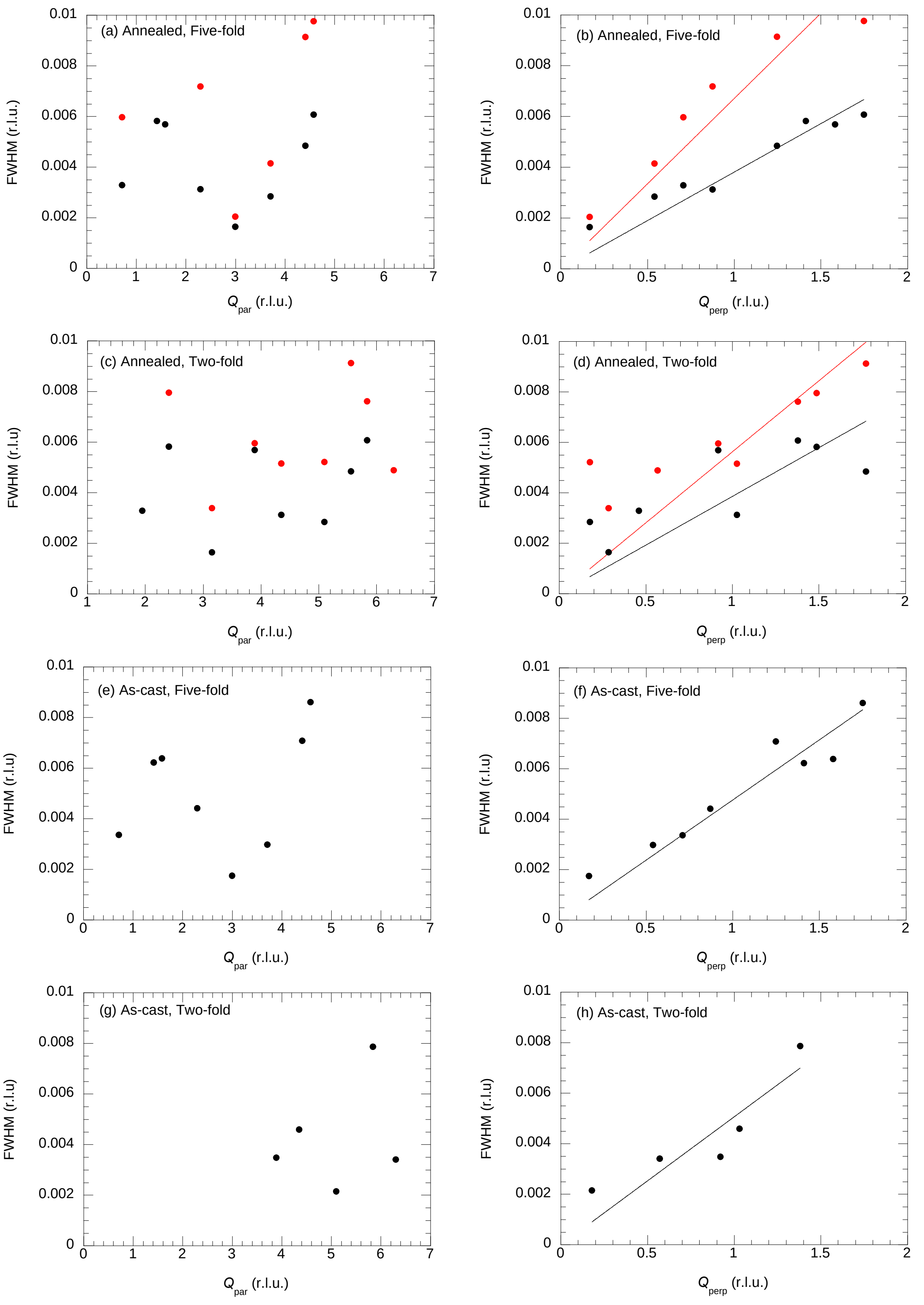

Figure S4 Evolution of the full-width at half-maximum (FWHM) of (a, b, e, g) five-fold and (c, d, g, h) two-fold reflections measured along the longitudinal (black) and transverse (red) directions for the (a-d) annealed and (e-h) as-cast i-$ScZn_{7.33}$ QC samples.

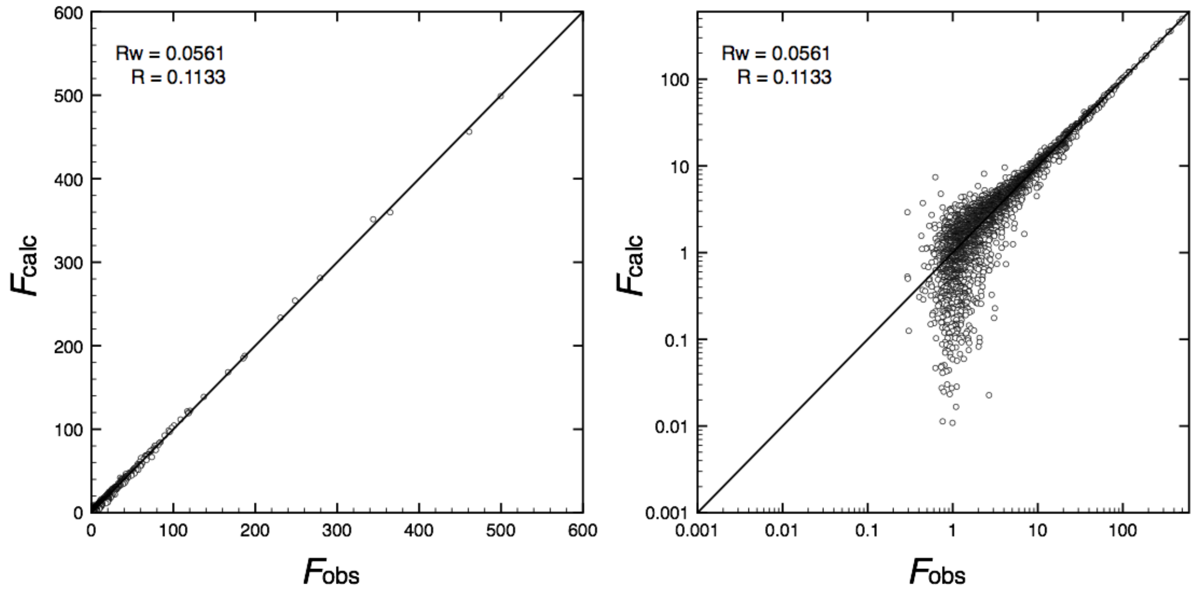

Figure S5 Normal and logarithmic F_{calc} versus F_{obs} plots of the structure refinement for the i-$ScZn_{7.33}$ QC. The icosahedron site was fully occupied by Sc and was fixed during the refinement (refinement 1). Unit weights are employed in the weighting scheme. $F_{calc}(000000) = 1877.69$.

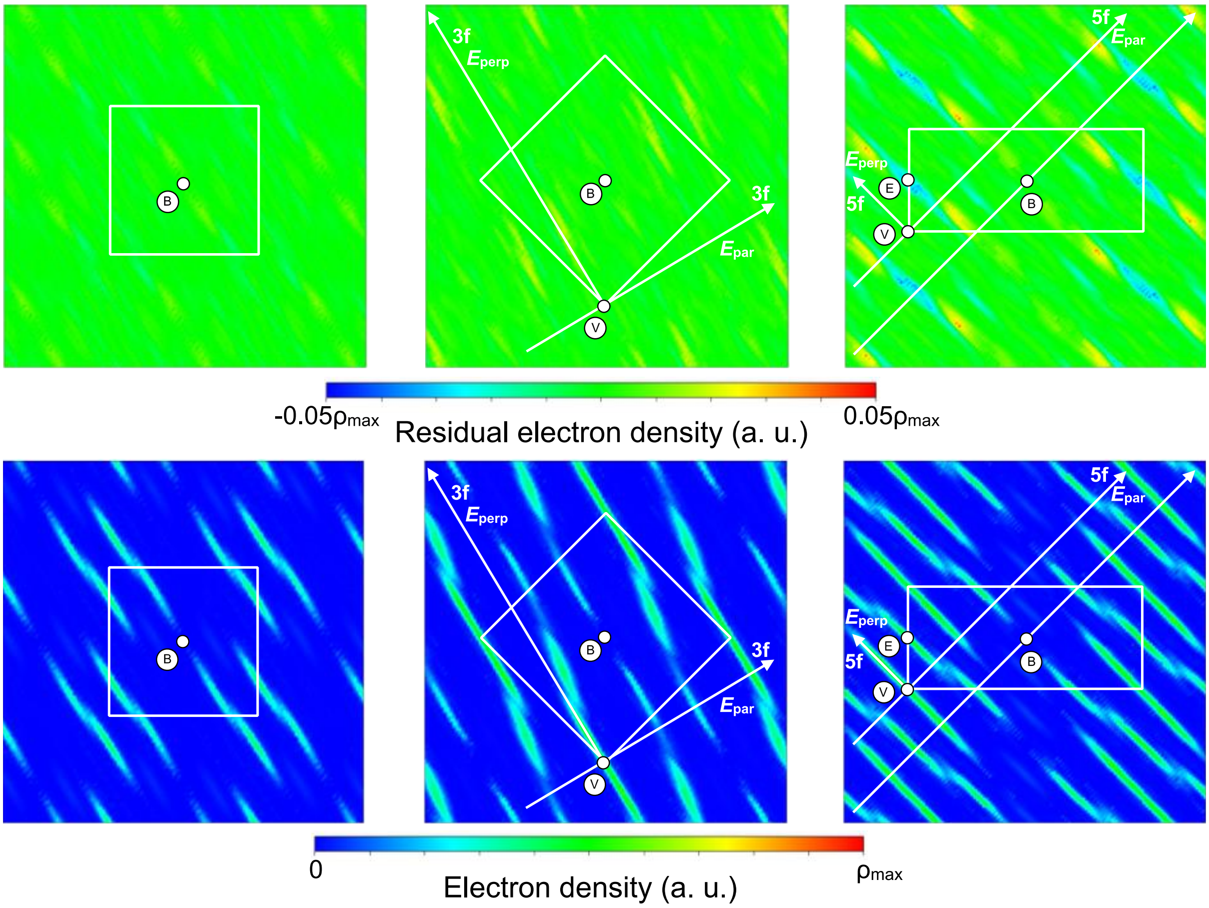

Figure S6 (top) Residual electron density distribution and (bottom) electron density distribution for the refined i-$ScZn_{7.33}$ QC (refinement 1). Left to right: 2-D sections containing two-fold, three-fold and five-fold axes both in E_{par} and E_{perp}.

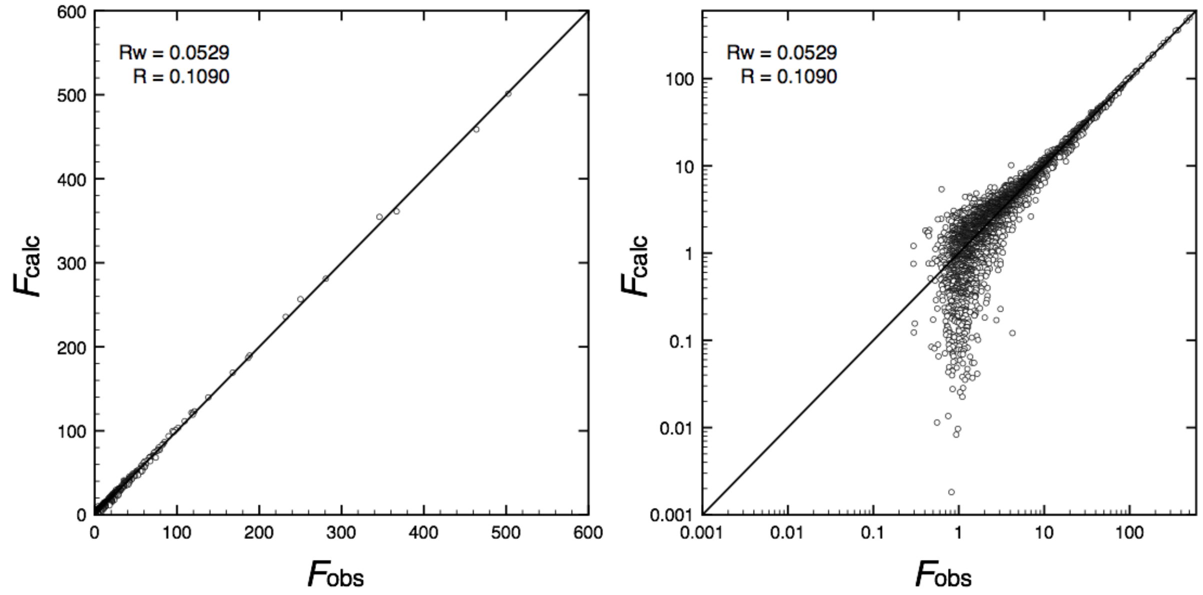

Figure S7 Normal and logarithmic F_{calc} versus F_{obs} plots of the structure refinement for the i-$ScZn_{7.33}$ QC. The occupancy of the icosahedron sites was refined with mix of Sc and Zn during the refinement (refinement 2). Unit weights are employed in the weighting scheme. $F_{calc}(000000)$ = 1878.82.

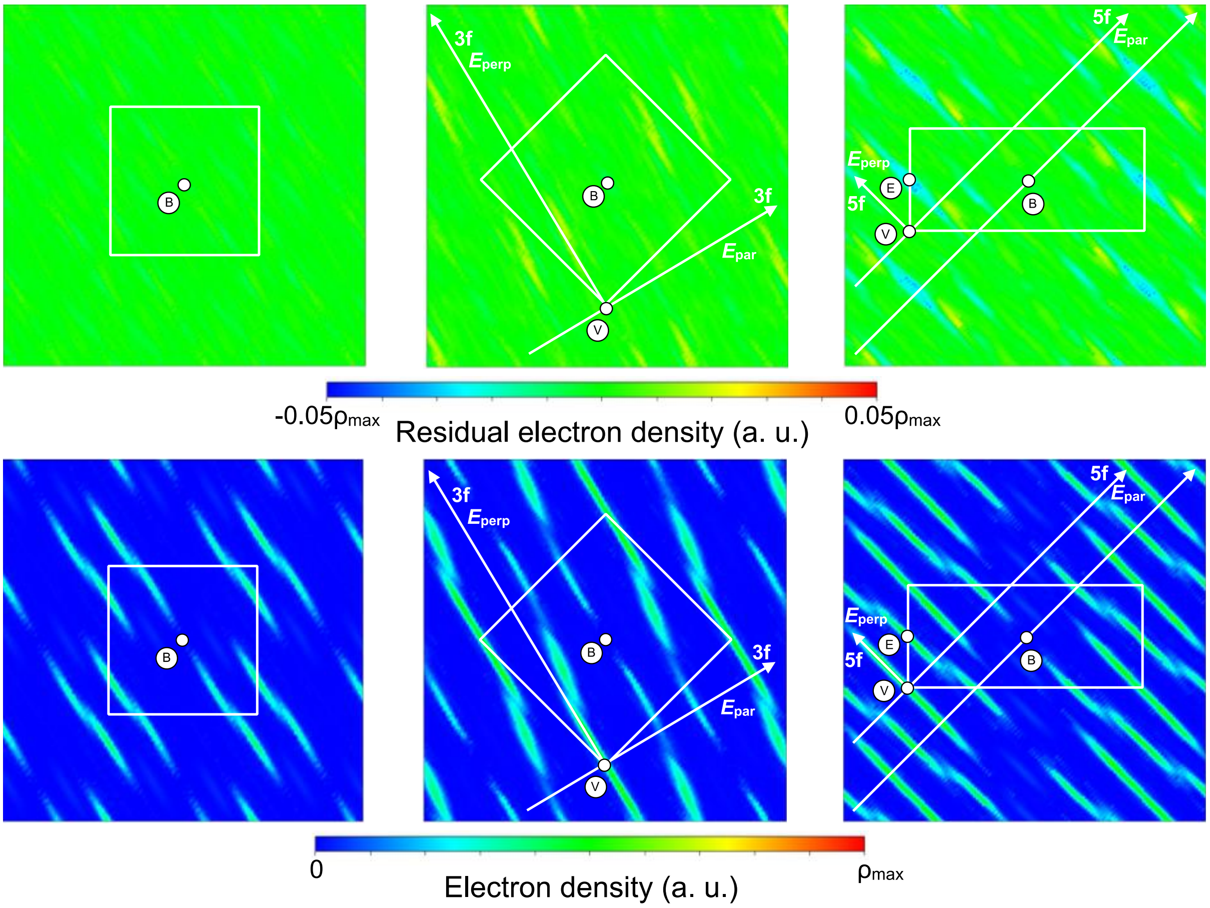

Figure S8 (top) Residual electron density distribution and (bottom) electron density distribution for the refined i-$ScZn_{7.33}$ QC (refinement 2). Left to right: 2-D sections containing two-fold, three-fold and five-fold axes both in E_{par} and E_{perp}.

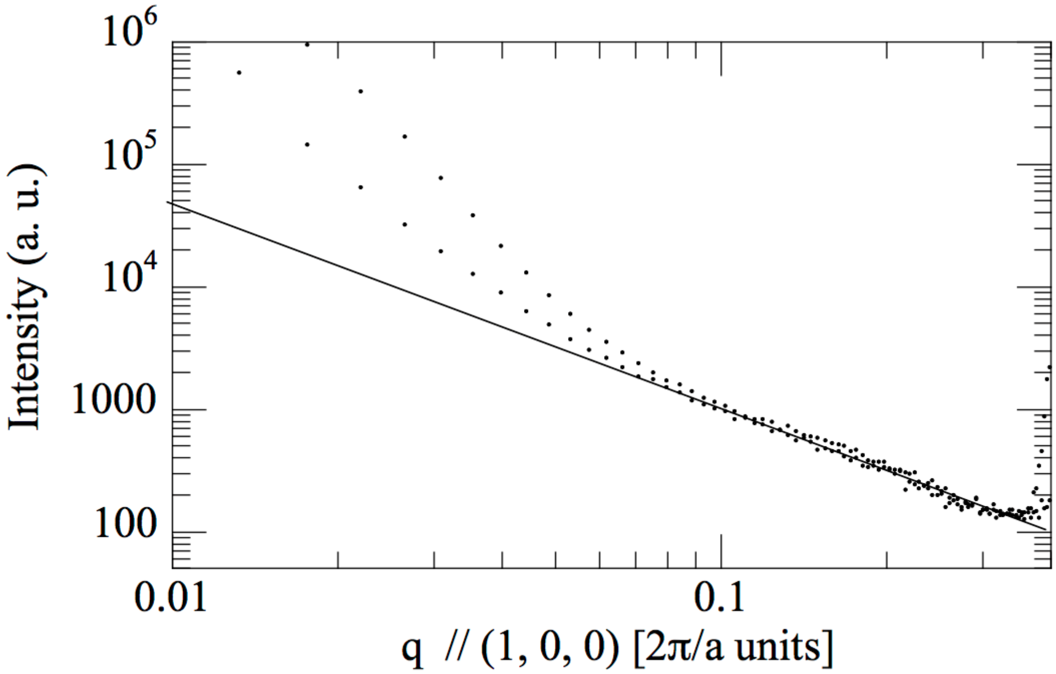

Figure S9 Intensity variation of the X-ray diffuse scattering with the distance q measured along a two-fold axis at the 18/29 five-fold reflection for the annealed i-$ScZn_{7.33}$ QC sample. The diffuse tail follows $1/q^2$ decay.

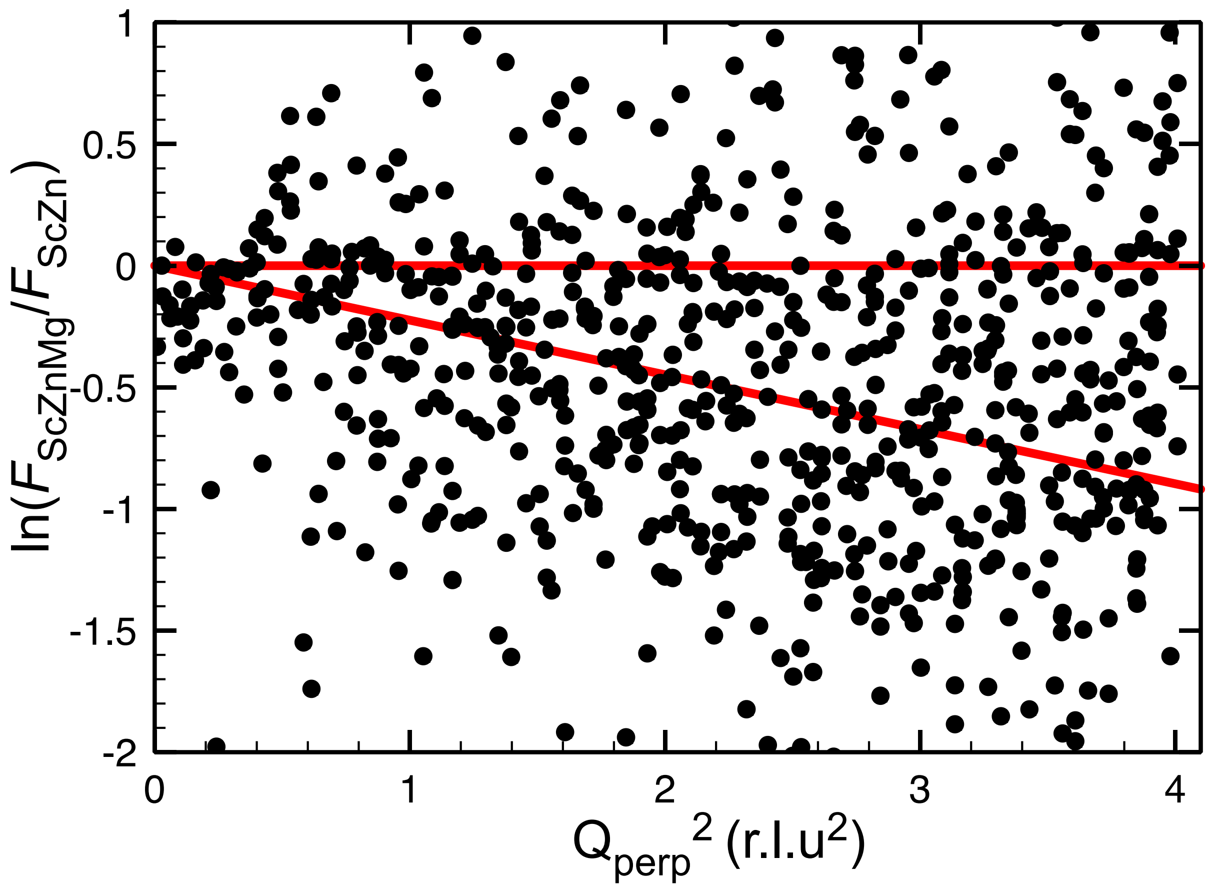

Figure S10 Logarithm plot of the ratio (F_{SZM}/F_{SZ}) as a function of Q_{perp}^2, where F_{SZM} and F_{SZ} stand for of the measured structure factors for i-Sc-Zn-Mg and i-$ScZn_{7.33}$ QCs respectively.

Table S1 Details of single-crystal X-ray data collection for i-$ScZn_{7.33}$ QC.

	Without attenuation	With medium attenuation	With maximum attenuation
Radiation source	Synchrotron		
Wavelength (Å)	0.48530		
Attenuation factor	1	50	1000
Detector	Agilent Atlas CCD		
Data collection method	Phi-scan		
Absorption correction	Multi-scan, SCALE3 ABSPACK		
T_{min}, T_{max}	0.85098, 1.00000	0.81877, 1.00000	0.78239, 1.00000
No. of measured and unique reflections	336227, 4750	20083, 478[a]	3729, 155 [a]
No. of measured and unique reflections ($I_{obs} \geq 3\sigma(I_{obs})$)	158205, 3819	20083, 478	3729, 155
Average redundancy	51.9	42.0	24.1
Completeness	100.00	100.00	100.00
No. of merged unique reflections	4778		
No. of merged unique reflections ($F \geq 3\sigma(F)$)	4057		
R_{int}	0.0928	0.0778	0.0881
Resolution limit (Å).	0.938 - 3.368	0.522 - 1.640	0.592 - 2.371

[a] Reflections $I_{obs} \geq 3\sigma(I_{obs})$ were counted as measured reflections.

Table S2 Crystallographic parameters and structural analysis information for i-$ScZn_{7.33}$ QC.

Crystal data		
Chemical formula	$Sc_{12\pm0.3}Zn_{88\pm0.3}$ (Canfield, 2010)	
Space group	$Pm\bar{3}\bar{5}$	
Temperature	293 K	
a_{ico} (Å)	5.021(3)	
Crystal form, size (μm)	Irregular, (40 × 40 × 60)	
	Refinement 1	Refinement 2
Weighting scheme	Unit weights	Unit weights
R [$\|F\| > 3\sigma(F)$]	0.1133	0.1090
Rw [$\|F\| > 3\sigma(F)$]	0.0561	0.0529
No. of parameters	202	199
Residual electron density	$\|\Delta\rho\| < 0.064\rho_{max}$	$\|\Delta\rho\| < 0.063\rho_{max}$
Refined composition	$ScZn_{7.38}$	$ScZn_{7.52}$
Point density (Å^{-3})	0.06364	0.06364

S4. Supporting crystallographic information file

A crystallographic information files (CIF) **ico-sczn.cif** contains atomic coordinates for 80 × 80 × 80 Å^3 regions for the refined atomic structure for the i-$ScZn_{7.33}$ QC with the mixed Sc/Zn icosahedron (refinement 2). The labels for the atomic positions correspond to those in Fig. S2. The z-axis corresponds to a five-fold direction. In addition, **center.cif** contains the coordinates of the RTH cluster centers in the same regions as above.

The **structure-factor.hkl** file contains structure factors for i-$ScZn_{7.33}$ QC with the mixed Sc/Zn icosahedron (refinement 2) with indices in both six indices by Cahn *et al.* (1986) , n_1, …, n_6, and that by Yamamoto (1996), h_1, …, h_6, mentioned in the section S2, together with N, M indices and cubic indices h/h’, k/k’ l/l’, by Cahn *et al.* (1986). The parallel and perpendicular components of the Bragg reflections, Q_{par} and Q_{perp}, are presented in reciprocal lattice units (r.l.u).

References

Cahn, J. W., Shechtman, D., & Gratias, D. (1986). *J. Mater. Res.* **1**, 13-26.

Boudard, M., de Boissieu, M., Janot, C., Heger, G., Beeli, C., Nissen, H., Vincent, H., Ibberson, R., Audier, M. & Dubois, J. M. (1992). J. Phys: Cond. Matter **4**, 10149-10168.

Cornier-Quiquandon, M., Quivy, M., Lefebvre, S., Elkaim, E., Heger, G., Katz, A. & Gratias, D. (1991). *Phys. Rev. B* **44**, 2071.

de Boissieu, M., Janot, C., Dubois, J. M., Audier, M. & Dubost, B. (1991). *J. Phys.: Condens. Matter* **3**, 1-25.

de Boissieu, M., Currat, R., & Francoual, S., in Physics of Quasicrystals, edited by T. Fujiwara and Y. Ishii (Elsevier Science, 2007), p. 107.

de Boissieu, M. (2008). *Philosophical Magazine*, **88**(13-15), 2295-2309.

de Boissieu, M. (2012). *Chemical Society Reviews*, 41(20), 6778-6786.

Elser, V. (1986). *Acta Cryst*. **A42**, 36-43.

Gratias, D., Puyraimond, F., Quiquandon, M., Katz, A., (2000). *Phys. Rev. B*, **63**, 024202

Henley, C. L. (1986). *Physical Review B*, **34**, 797-8165.

Horn, P. M., Malzfeldt, W., DiVincenzo, D. P., Toner, J., & Gambino, R. (1986). *Physical review letters*, **57**(12), 1444.

Ishii, Y. (1989). *Physical Review B*, **39**(16), 11862.

Ishii, Y. (1992). *Physical Review B*, **45**(10), 5228.

Janssen, T. (1991). *EPL (Europhysics Letter)s*, **14**(2), 131.

Janssen, T., Chapuis, G., & De Boissieu, M. (2007). Aperiodic crystals: from modulated phases to quasicrystals (No. LCR-BOOK-2007-001). Oxford: Oxford University Press, 2007.

Janssen, T. (1986). *Acta Cryst*. **A42**, 261-271.

Jarić, M. V., & Nelson, D. R. (1988). *Physical Review B*, **37**(9), 4458.

Kramer, P., & Neri, R. (1984) *Acta Clyst. A* **40**, 580

Lubensky, T. C., Socolar, J. E. S., Steinhardt, P. J., Bancel, P. A., & Heiney, P. A. (1986). *Phys. Rev. Lett.* **57**, 1440–1443.

Lubensky, T. C. (1988). *Introduction to Quasicrystals*, edited by M. Jaric, pp. 199-280. Academic Press.

Quiquandon, M. & Gratias , D. (2006). *Phys. Rev. B* **74**, 214205.

Socolar, J. E., & Wright, D. C. (1987). *Physical review letters*, **59**(2), 221.

Takakura, H., Gomez, C. P., Yamamoto, A., De Boissieu, M. & Tsai, A. P. (2007). *Nat Mater*, **6**(1), 58–63.

Widom, M. (1991). *Philosophical Magazine Letters*, **64**(5), 297-305.

Yamamoto, A. (1992). *Physical Review B*, **45**, 5217-5227.

Yamamoto, A. (1996). *Acta Cryst.* **A52**, 509-560.

Yamamoto, A., Takakura, H. & Tsai, A. P. (2003). *Phys. Rev. B* **68**, 94201-94201-94213.

Yamamoto, A. (2008). *Science and Technology of Advanced Materials*. **9**, 013001.
